# Constructing pathways for glycine betaine in cross-domain ecological restoration: a bibliometric analysis

**DOI:** 10.7717/peerj.21523

**Published:** 2026-08-21

**Authors:** Zhe Wu, Junbao Li, Zhilin Ma, Jingle Zhou, Honggang Huang

**Affiliations:** 1 Key Laboratory of State Forestry Administration on Soil and Water Conservation & Ecological Restoration of Loess Plateau, Shaanxi Academy of Forestry, Xi’an, Shaanxi Province, China; 2 Research Institute of Wetland and Grassland Protection, Shaanxi Academy of Forestry, Xi’an, Shaanxi Province, China; 3 Central South Academy of Inventory and Planning of NFGA, Changsha, Hunan province, China; 4 Institute of Ecological Restoration and Industrial Development, Shaanxi Academy of Forestry Sciences, Xi’an, Shaanxi Province, China

**Keywords:** Glycine betaine, Grassland ecological restoration, Technology transfer, Conceptual model, Bibliometrics, Social-ecological values

## Abstract

**Background:**

Grassland ecosystems play a critical role in global ecological security and sustainable development. However, they are experiencing continuous functional decline due to multiple stresses under climate change. Glycine betaine (GB) has been systematically studied for enhancing stress tolerance in agricultural crops. Nevertheless, a knowledge gap exists regarding its application in the restoration of degraded grasslands. What social-ecological values might emerge from addressing this gap?

**Methods:**

To address these questions, this study conducted a systematic bibliometric analysis of GB research from 1980 to 2023. The analysis was based on 295 core publications: 132 from the China National Knowledge Infrastructure and 163 from the Web of Science Core Collection. Tailored query strategies were employed in both databases to capture studies on exogenous GB application in plants. After rigorous screening to exclude irrelevant topics, the final corpus was analyzed using VOSviewer and CiteSpace. Analyses included keyword evolution, co-occurrence networks, research trajectory mapping, and collaboration pattern visualization.

**Results:**

The analysis reveals a complementary global research landscape. International studies exhibit a “mechanism-oriented” trajectory, while Chinese research demonstrates an “application-driven” trajectory. However, research subjects are overwhelmingly concentrated on agricultural crops. Studies on keystone grassland species are nearly absent. Research settings are largely confined to controlled environments, lacking field validation and ecosystem-level assessments. This structural gap provides an empirical basis for further investigation. Building on it, this study adopts a “technology-ecology-society” integrated perspective. It proposes a conceptual five-node cyclic model: Technology → Ecology→ Well-being → People→ City→ Technology. The model redefines GB from a static “technological tool” into a dynamically evolving agent within the social-ecological system. It outlines a three-phase evolutionary pathway: Emergency Response, Functional Restructuring, and Model Transition.

**Conclusion:**

The core value of the model lies in articulating that the migration of GB technology from agriculture to grassland restoration could initiate a virtuous cycle. Rural and urban residents respectively gain the economic and ecological values they currently lack. Enhanced well-being drives the stabilization and agglomeration of human resources. Urban prosperity reinforces technological innovation. This model serves as a heuristic tool. It provides a theoretical framework for the targeted migration of GB technology into grassland ecosystem restoration. It also offers a transferable conceptual model for other agricultural technologies entering ecosystem restoration domains.

## Introduction

Grassland ecosystems cover approximately 27% of the Earth’s terrestrial area (Sun et al., 2022; Bardgett et al., 2021). They play a crucial role in maintaining biodiversity, conserving soil and water (Avishek & Kumar, 2021), supporting livestock industry development (Wang & Wang, 2019), and promoting ecological tourism (Zhu et al., 2023). However, with the intensifying effects of climate change, grasslands face severe challenges such as aridification, salinization, and heavy metal contamination (Hu et al., 2023). Studies from 2000 to 2023 indicate that drought reduces grassland biomass, net primary productivity, microbial carbon and nitrogen contents, and soil respiration intensity. In contrast, soil organic carbon, total nitrogen, total phosphorus, and nitrate nitrogen show an increasing trend (Liu et al., 2023). In response to these challenges, traditional management practices such as grazing prohibition, rotational grazing, reseeding, and fertilization have revealed limitations (Slodowicz et al., 2023). Therefore, integrating new scientific findings is urgently needed.

In this context, glycine betaine (GB) demonstrates significant application potential. GB is a quaternary ammonium alkaloid (Chen et al., 2025a; Chen et al., 2025b). It maintains cellular osmotic balance, activates antioxidant enzyme systems (Khalid et al., 2024), protects photosynthetic structures from photoinhibition, and improves light use efficiency (Badawy et al., 2024). Current research on exogenous GB primarily focuses on enhancing crop stress resistance (Basit et al., 2025). For instance, under drought conditions, treatment with three mmol/L GB significantly increased maize shoot and root biomass by 51.55% and 42.70%, respectively. Under salt stress, it enhanced wheat tolerance by modulating intracellular ion homeostasis (Farooq et al., 2024). In cadmium-contaminated environments, GB could reduce cadmium uptake through antagonistic effects (Islam et al., 2009). Although the physiological functions of GB have been well established, existing research is heavily biased toward growth stages such as seed germination (Su et al., 2024; Zhang et al., 2022; Ahmed et al., 2019). Few studies extend to field validation and ecosystem-level application assessments. This research imbalance severely limits the technology’s promotion and application in real-world ecological restoration scenarios.

Furthermore, although the synthesis mechanisms and physiological functions of GB are well understood in agricultural research, a significant knowledge gap exists in its migration to grassland ecological restoration. First, research objectives differ fundamentally. Agricultural studies focus on increasing yield (Zhang et al., 2020a; Zhang et al., 2020b), whereas ecological restoration requires balancing multiple goals, including ecosystem stability, production functions, and service values. Second, existing research is largely conducted under idealized controlled conditions (Liu et al., 2025). This makes it difficult to address complex field environments. Third, the research subjects are predominantly artificially cultivated species like crops, forage grasses, and turfgrasses (Shemi et al., 2021). Limited attention is given to native vegetation. Fourth, the evaluation system exhibits significant limitations. The prevailing evaluation framework privileges plant-level metrics over ecosystem-scale dynamics (Wang et al., 2023). Therefore, systematically reviewing the research progress on GB has become an urgent necessity.

Based on the aforementioned analysis, this study aims to answer two interrelated questions:

(1) Does a genuine gap exist between exogenous GB research in agriculture and the needs of grassland ecological restoration? What evidence supports this?
(2) What values might emerge from filling this gap? Beyond basic scientific value, are there broader social-ecological values?

To address these questions, this study first employs bibliometric methods to systematically review exogenous GB research from 1980 to 2023. It quantitatively maps the research landscape in this field and objectively identifies structural gaps in existing studies. On this basis, this study adopts a “technology-ecology-society” integrated perspective to construct a conceptual model that explores the potential social-ecological values of transferring GB technology to grassland restoration. These values span multiple dimensions, including technology, ecology, economy, and society. The aim is to provide theoretical inspiration for future empirical research and interdisciplinary inquiry.

## Basic research

### Fundamental mechanisms

The mechanisms by which GB enhances plant stress tolerance have formed a relatively complete understanding at the molecular, cellular, and physiological levels. These mechanisms are primarily reflected in three synergistic aspects: osmotic adjustment, antioxidant defense, and photosynthetic protection.

#### Osmotic adjustment mechanism

As a highly compatible osmolyte, the core function of GB lies in maintaining cellular water and ion homeostasis (Basit et al., 2025). First, it accumulates abundantly in the cytoplasm, acting as an osmotic buffer to balance ion concentrations in the vacuole (Annunziata et al., 2019). This effectively prevents cellular dehydration and maintains cell turgor pressure and structural integrity (Chen et al., 2025a; Chen et al., 2025b; Burg, 1995). Second, GB can mitigate ion toxicity by stabilizing membrane systems and inhibiting the accumulation of Na^+^ in the cytoplasm (Khan, Khan & Ahmad, 2016). Furthermore, it synergizes with endogenous osmolytes such as soluble sugars to collectively protect enzyme activity and protein spatial structure, maintaining normal metabolic function (Singh et al., 2025). Consequently, although GB does not directly participate in ion transmembrane transport, it significantly enhances plant adaptability to salt stress (Singh et al., 2025).

#### Antioxidant defense mechanism

Under oxidative stress, GB activates the enzymatic antioxidant system to establish a core defense barrier (Basit et al., 2025). Its primary contribution lies not in directly scavenging reactive oxygen species. Instead, it significantly upregulates the activities of key antioxidant enzymes including superoxide dismutase, catalase, ascorbate peroxidase, and glutathione reductase (Kumar et al., 2025). This achieves systematic reactive oxygen species scavenging (Basit et al., 2025). GB works synergistically with other antioxidative substances like proline to jointly suppress membrane lipid peroxidation (Lamlom et al., 2025). This effectively protects the integrity of the membrane system.

#### Photosynthetic protection mechanism

GB provides particularly prominent protection for photosynthetic structures (Annunziata et al., 2019). First, it directly stabilizes the photosystem II supercomplex. This prevents protein denaturation and aggregation under stress conditions such as high temperatures. Second, it safeguards thylakoid membrane integrity, ensuring the normal operation of the electron transport chain (Li et al., 2024). Additionally, it significantly enhances light energy conversion efficiency and photoprotective capacity (Singh et al., 2025), while synergizing with the antioxidant system to mitigate oxidative damage caused by photosynthesis and respiration (Ayub et al., 2022).

### Technological foundations

#### Genetic engineering technology

The genetic engineering technology for GB primarily relies on the transformation of two key genes involved in its synthesis pathway: betaine aldehyde dehydrogenase (BADH) and choline monooxygenase (CMO) (Li et al., 2025). This technology has demonstrated high versatility, with BADH and CMO genes having been successfully introduced into various important crops such as tobacco, rice, wheat, and cotton (Zhang et al., 2020a; Zhang et al., 2020b; Wang et al., 2010; Zhang et al., 2019; You et al., 2019). Although genetic engineering confers endogenous GB synthesis capacity to plants, its application in open environments such as grassland ecological restoration faces significant limitations.

#### Exogenous application technology

In contrast to genetic engineering, the exogenous GB application is a non-genetically modified management approach. It is characterized by low cost, simple operation, and rapid effectiveness (Yang et al., 2022a; Yang et al., 2022b). These features make it highly promising for translation to grassland ecological restoration. This technology is primarily implemented through three methods: foliar spraying, root irrigation, and seed soaking (Baroi et al., 2024). Foliar spraying is the mainstream application method due to its operational simplicity and high absorption efficiency. Surfactants are often added to further enhance utilization efficiency (Subbarao et al., 2001). From the perspective of application stage, existing research covers multiple growth stages such as seed germination, seedling development, and adult plant stress resistance (Wang et al., 2014). From the perspective of dose–response relationships, optimal GB concentrations vary among crops (Table S1). Overall, suitable GB application concentrations generally range from 5 to 200 mmol/L. Notable species specificity exists: grain crops (*e.g.*, rice, maize) typically respond to lower concentrations (0.5–10 mmol/L), while cash crops and fruit trees (*e.g.*, apple, pomegranate) can tolerate higher concentrations (up to 200 mmol/L). These dose–response relationships provide important references for concentration screening for keystone grassland species.

## Materials & Methods

### Data sources

The data for this study were sourced from the China National Knowledge Infrastructure (CNKI) and the Web of Science Core Collection (WoS). The search cutoff date was set to December 31, 2023. In CNKI, an advanced search was conducted within the Botany and Agricultural Science & Technology categories. The query used was “SU% = ‘甜菜碱’ AND (SU% = ‘添加’ OR SU% = ‘外源’)”, yielding 206 journal articles (甜菜碱: glycine betaine; 添加: addition; 外源: exogenous).

In WoS, the search query was “TS = (betaine) AND TS = (addition OR exogenous) NOT WC = (Zoology OR Microbiology) AND WC = (Plant Sciences OR Ecology OR Agronomy OR Horticulture OR Environmental Science)”. This retrieved 632 publications.

### Literature screening

These 632 articles were imported into reference management software. They underwent initial screening by reading titles and abstracts. The inclusion criteria for initial screening were studies addressing the effects of exogenous GB application on plant growth. These included physiological processes such as seed germination, seedling development, and stress tolerance in adult plants. Exclusion criteria included: (1) tissue culture studies (*e.g.*, callus induction, suspension cell culture); (2) studies involving detached organs (*e.g.*, fruits, flowers, seed sections), which are more commonly associated with GB applications in low-temperature storage of fruits and vegetables; (3) studies investigating changes in endogenous GB content under the influence of other exogenous additives (*e.g.*, salicylic acid, abscisic acid); (4) non-original research (*e.g.*, reviews, conference abstracts, theses); (5) articles for which full text could not be obtained or data were incomplete.

**Figure 1:**
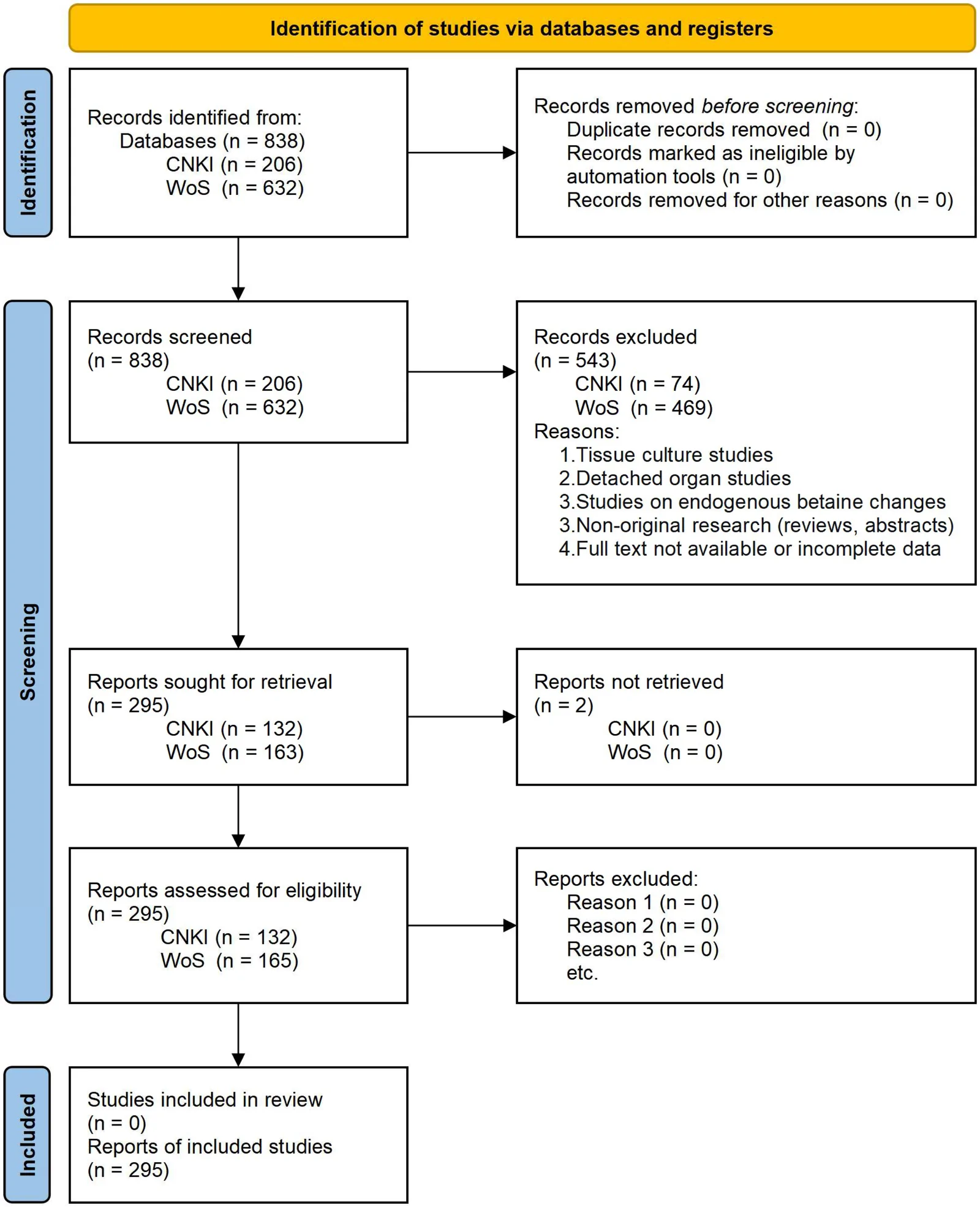
PRISMA flow diagram of the literature screening and study selection process. Note: The flow diagram shows the number of records identified from WoS and CNKI databases, the number excluded during screening with reasons, and the final number of included studies (*n* = 295).

Following screening by title, abstract, and full text, 132 CNKI articles and 163 WoS articles were ultimately included. This resulted in a total of 295 valid articles for subsequent analysis (Fig. 1).

### Data processing and analysis

After completing the literature screening, the 132 CNKI articles were first converted to a CiteSpace-compatible format. This ensured compatibility with WoS data. Reference information for CNKI articles was manually supplemented to ensure data completeness. The processed CNKI data were then merged with the 163 WoS articles. This constructed a standardized database containing fields such as authors, affiliations, titles, journals, publication dates, keywords, and references. The database encompassed a total of 295 articles.

For data analysis, this study employed VOSviewer and CiteSpace for integrated processing. SCImago Graphica was used to enhance the visualization of institutional collaboration networks. CiteSpace was primarily used for keyword co-occurrence and clustering analysis, keyword burst detection, document co-citation analysis, and timeline evolution analysis. Parameter settings were as follows: time slices from 1980 to 2023, with each slice representing a 3-year interval; node types selected were “keyword” and “cited reference”; the threshold was set to include nodes with a frequency ≥2 within each time slice for network analysis (Li & Chen, 2016). VOSviewer was used for country/institution collaboration networks, keyword co-occurrence density visualization, and author co-citation analysis. Specific parameter settings were as follows: counting method was set to full counting; minimum co-occurrence frequency was set to 5; normalization method was association strength; clustering resolution parameter was set to 1.0 (van Eck & Waltman, 2010). On this basis, the institutional collaboration network output from VOSviewer was imported into SCImago Graphica (Chen et al., 2025a; Chen et al., 2025b). This generated institutional distribution maps to visually illustrate the regional patterns of research capacity.

## Results

### Evolution and comparison of research hotspots

Based on keyword evolution analysis, the field of exogenous GB research reveals two distinct development pathways. The international “mechanism-deepening” path centers on mechanistic exploration. The domestic “application-expanding” path is driven by practical needs. Although their objectives differ, they are functionally complementary and together constitute the complete knowledge landscape of this field.

The international “deepening” trajectory represents a cognitive process that progressively advances from macroscopic physiological phenomena to microscopic molecular networks. This process can be divided into four stages: the Emergence Phase (1982-1995) focused on ion transport and proline exploration in barley and wheat under salt stress; the Start-up Phase (1996–2009) expanded stress types to include drought, with antioxidant enzyme systems and osmotic adjustment becoming core mechanistic research topics; Outbreak Phase I (2010–2016) shifted toward agricultural applications, incorporating crop yield and photosynthetic parameters into the analytical framework; Outbreak Phase II (2017–2023) further deepened research on combined stresses (Table 1). Interactions between metal stress and salt/drought stress emerged as new research hotspots, and techniques such as chlorophyll fluorescence and photosystem dynamics monitoring were widely applied. Overall, this trajectory represents a process of progressive deepening, moving inward and simultaneously expanding cognitive horizons.

**Table 1:** Evolution of international exogenous GB research hotspots (1982–2023).

**Stage**	**Time**	**High-frequency keywords (frequency)**	**Research focus**
Emergence Phase	1982–1995	barley (3), salt stress (2), wheat (1), pyruvate kinase (1), ion transport (1), halophyte (1), proline (1), betaine aldehyde dehydrogenase (1)	Core Crops: Barley and wheat. Core Mechanisms: Preliminary exploration of ion transport and proline under salt stress. Technical Characteristic: Primarily based on physiological index measurements.
Start-up Phase	1996–2009	drought stress (10), salt stress (8), proline (8), antioxidant enzymes (7), osmotic adjustment (5), photosynthesis (3), rice (3), chlorophyll fluorescence (3), cold tolerance (3)	Expansion of Stress Types: Equal emphasis on drought and salt stress. Breakthrough in Molecular Mechanisms: Antioxidant enzyme systems and osmotic adjustment become central. Crop Diversity: Rice and maize are incorporated into the research system.
Outbreak Phase 1	2010–2016	drought stress (13), antioxidant enzymes (12), salt stress (10), photosynthesis (8), proline (7), yield (5), maize (5), antioxidant defense (4)	Agricultural Application Orientation: Focus on crop yield. Research on Stress Interactions: Mechanisms linking high temperature and oxidative stress. Indicator of Technological Integration: Combined analysis of photosynthesis parameters and stress tolerance.
Outbreak Phase 2	2017–2023	salt stress (26), photosynthesis (18), drought stress (16), yield (12), proline (11), chlorophyll (11), ionic homeostasis (8), antioxidant enzyme (8), metal stress (6), cotton (5)	Deepening of Combined Stress Research: Cross-study of metal stress with salt/drought stress. Refinement of Photosynthetic Mechanisms: Dynamic monitoring of chlorophyll and photosystems. Species Diversification: Significant increase in the proportion of economic crops (*e.g.*, cotton, cucumber, tomato).

In contrast, the domestic “application-expanding” trajectory reflects the horizontal expansion of application scenarios and the precise upgrading of solutions. It demonstrates a distinct “problem-solving” orientation. The Start-up Phase (1996–2009) primarily focused on membrane lipid peroxidation responses in wheat seedlings under salt stress; Outbreak Phase I (2010–2016) expanded stress types to include drought and low temperature (Table 2). Research subjects extended from grain crops to cash crops such as pepper, cotton, and tobacco. The research stage shifted from seedling growth to seed germination; Outbreak Phase II (2017–2023) exhibited two major characteristics. First, combined stress research deepened. Heavy metal stress began to be analyzed in conjunction with salt, drought, and low temperature stresses. Second, regulatory approaches were upgraded. The combined application of signaling molecules such as salicylic acid and exogenous calcium increased significantly. The research focus shifted from “phenomenon description” to exploration of “alleviation effects”.

**Table 2:** Evolution of domestic exogenous GB research hotspots in China (1996–2023).

**Stage**	**Time**	**High-frequency keywords (frequency)**	**Research focus**
Emergence Phase	1982–1995	——	——
Start-up Phase	1996–2009	Salt stress (18), Wheat (8), Seedling (8), Membrane lipid peroxidation (1)	Stress Type: Salt stress, with membrane lipid peroxidation serving as an early marker of oxidative damage. Research Subject: Wheat as the representative crop. Technical Methods: Primarily based on measurement of basic physiological indices (*e.g.*, membrane lipid peroxidation). Research Stage: Seedling growth.
Outbreak Phase 1	2010–2016	Drought stress (14), Low temperature stress (11), Salt stress (10), Physiological characteristics (9), Antioxidant enzymes (5), Germination (5)	Stress Types: Equal emphasis on drought, low temperature, and salt stress. Research Subjects: Economic crops such as pepper, cotton, and tobacco. Mechanistic Deepening 1: Antioxidant enzyme systems become a core research focus. Mechanistic Deepening 2: Osmotic adjustment and photosynthesis begin to be linked to stress tolerance. Research Stage: Seed germination stage.
Outbreak Phase 2	2017–2023	Physiological characteristics (20), Salt stress (18), Drought stress (15), Seed germination (13), Low temperature stress (12), Seedling growth (11), Salicylic acid (9), Maize (8), Heavy metal stress (6), Exogenous calcium (6)	Combined Stresses: Cross-analysis of salt, drought, low temperature, and heavy metal stresses. Research Subjects: Various crops including maize, alfalfa, and rice. Research Deepening 1: Increased application research on signaling molecules such as salicylic acid and exogenous calcium. Research Deepening 2: Research focus shifts towards “alleviation effects”. Research Stage: From seed germination to seedling growth.

**Figure 2:**
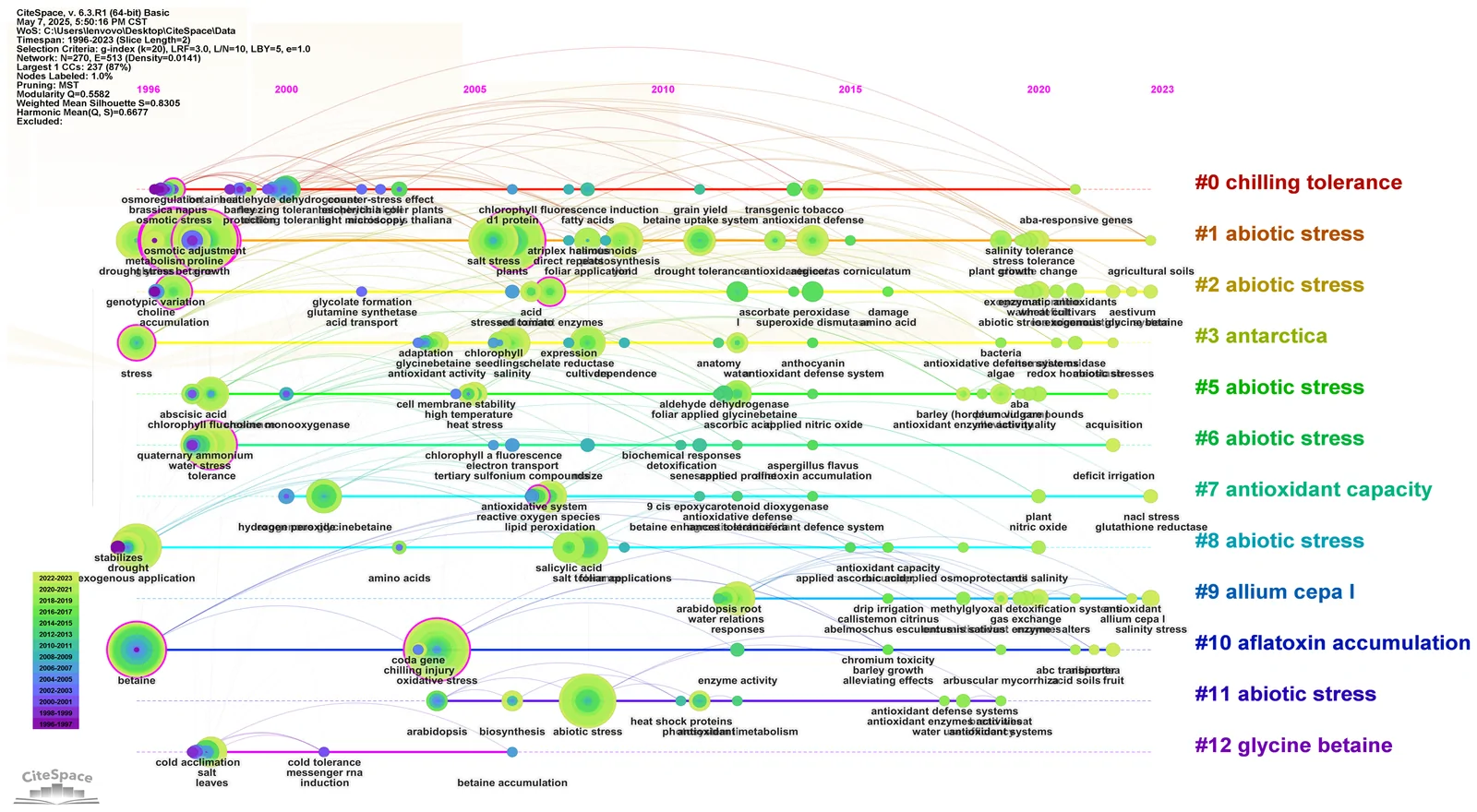
Keyword clustering timeline of exogenous GB research. Note: The figure presents a timeline view of keyword co-occurrence clusters generated by CiteSpace. The horizontal axis represents time (1980-2023, with 3-year slices), and the vertical lists cluster numbers. Each horizontal line corresponds to a keyword cluster, with nodes representing individual keywords. Node size reflects keyword frequency, and node color indicates the time slice in which the keyword first appeared. Clusters are labeled using the log-likelihood ratio (LLR) algorithm. Early clusters (*e.g.*, Cluster 0, Cluster 1) focus on the correlation between exogenous glycine betaine (GB) application and plant stress tolerance, while later clusters (*e.g.*, Cluster 2: Induced Oxidative Stress) reflect a shift toward mechanistic exploration of antioxidant enzyme activity and cellular signaling pathways.

These evolutionary trends are further corroborated by keyword clustering analysis (Fig. S1). Early studies (Cluster 0 and Cluster 1) focused on the core question: the relationship between GB and stress tolerance. By observing changes in plant characteristics after exogenous application, these studies quantitatively described the correlation between the two (Fig. 2). With the widespread adoption of molecular biology techniques, the research question shifted toward the functional expression and mechanisms of GB. Mechanism-oriented research, represented by Cluster 2 (Induced Oxidative Stress), became mainstream. The focus shifted to how GB influences antioxidant enzyme activity and cellular metabolic pathways through signal regulation. This shifted research from macroscopic descriptions to microscopic mechanisms.

Current research exhibits a clear application orientation, centering on strategies for using GB to mitigate stress. On the one hand, basic research is expanding into agricultural applications. It moves from understanding nature to intervening in and optimizing agricultural production systems. This includes studying different application methods (foliar application, root irrigation), combinations with other protective agents, and targeted protocols for specific crop-stress combinations. On the other hand, research approaches are deepening from exogenous supplementation to genetic modification. Genetic techniques are used to optimize the GB synthesis pathways in crops. This creates new varieties with higher intrinsic stress tolerance.

### Collaboration networks and knowledge foundation

Research on exogenous GB exhibits a multipolar pattern in terms of global research strength. It centers around the United States, China, and Pakistan (Fig. S2). The United States holds the highest number of English-language publications (41 articles). These are primarily supported by institutions such as Oregon State University. Research directions focus on the photosynthetic mechanisms of GB and crop stress tolerance. Following closely, China has published 36 English-language articles. Strong research capabilities are demonstrated by institutions including the Chinese Academy of Agricultural Sciences, Northwest A&F University, and Shandong Agricultural University. Chinese research places greater emphasis on seed germination, seedling growth, and responses to heavy metal stress (*e.g.*, cadmium). Pakistan, as a significant participant, has published 34 English-language articles. These are led mainly by the University of Agriculture, Faisalabad. The research focuses on GB under drought stress (Table 3). International collaboration is particularly active. Scholars such as Ashraf, Yang X.H., and Chen T.H.H. demonstrate high collaboration intensity (Table S2 & Fig. S3).

**Table 3:** Country and institutional distribution of exogenous GB publications.

**Country**	**Number of English papers**	**Organization**	**Total number of papers**	**Number of Chinese papers**
USA	41	University of Agriculture, Faisalabad	19	–
China	36	Chinese Academy of Agricultural Sciences	12	7
Pakistan	34	Northwest A&F University	11	7
Iran	13	Shandong Agricultural University	11	2
Egypt	11	Southwest University	9	8
Japan	10	Gansu Agricultural University	9	4
Türkiye	10	Government College University, Faisalabad	7	–
Saudi Arabia	9	Islamic Azad University	7	–
India	9	King Saud University	6	–
Spain	6	Oregon State University	6	–

Through knowledge graph analysis, this study reveals that the systematic review by Ashraf & Foolad (2007) established a comparative research framework for GB and proline. This has become a core component of the domain’s knowledge base (Fig. S4). At the level of physiological mechanisms, Pollard & Wyn Jones (1979) enzymatic studies and Coughlan & Heber (1982) research on cryoprotective mechanisms together elucidate the fundamental physiological functions of GB. They form important knowledge starting points for Cluster 0 (Environmental Stress Response) and Cluster 1 (Core Mechanisms of GB) (Fig. S5). Domestic research highlights that in-depth investigations by Liang & Luo (1995) and Liang et al. (1996) on betaine aldehyde dehydrogenase, studies by Guo et al. (2004) on wheat salt resistance, and research by Li et al. (2004) on cucumber cold tolerance have shaped a distinctive applied research direction. This directly led to subsequent focused attention on drought stress and seedling growth (Table 4).

**Table 4:** Major researchers and their representative contributions.

**Author**	**Papers number**	**Cooperation intensity**	**Representative results**
Ashraf M.	14	40	Iqbal, Ashraf & Ashraf (2008), Glycinebetaine, an osmolyte of interest to improve water stress tolerance in sunflower (*Helianthus annuus* L.): water relations and yield
Yang X.H.	6	26	Yang & Lu (2006). Effects of exogenous glycinebetaine on growth, co_2_ assimilation, and photosystem II photochemistry of maize plants
Chen T.H.H.	6	25	Park, Jeknic & Chen (2006). Exogenous application of glycinebetaine increases chilling tolerance in tomato plants
Hua Z.R.	6	4	Hua, Liu & Li (2022). Effect of exogenous betaine on physiological characteristics of *Platycodon grandiflorum* seedlings under salt stress [In Chinese]
Ali S.	4	22	Ali & Ashraf (2011). Exogenously applied glycinebetaine enhances seed and seed oil quality of maize (*Zea mays* L.) under water deficit conditions
Wang Y.N.	4	14	Wang et al. (2007). Effects of exogenous betaine on physiological responses of peach tree under water stress [In Chinese]
Ba Q.S.	4	13	Ba et al. (2019). Effects of root-applying glycine betaine on reactive oxygen species metabolism ofwheat roots under lead stress [In Chinese]
Li G.P.	4	13	Chen et al. (2020). Effects of exogenous glycine betaine on rwactive oxygen species metabolism in super black waxy maize under cadmium stress [In Chinese]
Pehu E.	4	10	Mäkelä et al. (1996). Uptake and translocation of foliar-applied glycinebetaine in crop plants
Peltonensainio P.	4	10	Agboma et al. (1997). Exogenous glycinebetaine enhances grain yield of maize, sorghum and wheat grown under two supplementary watering regimes

Analysis of the knowledge network reveals that international and domestic research has not developed in isolation. Instead, they are interconnected through key nodes. They form a landscape with distinct characteristics and complementary strengths. The two are linked through key literature as connecting nodes. This establishes close connections between internationally common physiological indicators (*e.g.*, photosynthesis) and molecular mechanisms with domestic research characteristics (Fig. S6). However, when it comes to broader themes such as climate change adaptation and responses to combined stresses, the deep integration between domestic and international research remains insufficient. This research gap is precisely one of the key bottlenecks hindering the “cross-domain technology transfer” from agriculture to ecological restoration.

### Bottleneck analysis

Extending exogenous GB from crop stress tolerance research to natural grassland ecological restoration reveals three structural gaps. These gaps exist beneath the surface of abundant achievements. First, the research subjects are concentrated on crops, while research on keystone native species remains understudied, such as *Stipa* and *Leymus chinensis*. This disciplinary path dependency leaves the technology transfer lacking the most fundamental baseline understanding. Second, research settings are dominated by idealized controlled environments. Field validation is absent. This makes it difficult to evaluate the practical efficacy and ecological adaptability of research outcomes. Third, mechanistic understanding remains confined to the individual physiological level. Multi-scale ecological integration is insufficient. This inadequacy fails to support the ecosystem-level intervention required for grassland restoration.

The combination of these three gaps forms a fundamental obstacle. The research logic of “crop individual to yield” in agricultural contexts cannot be directly applied to the multi-layered restoration goal of “species-community-ecosystem”. To advance the exogenous GB technology toward the practical application of grassland ecological restoration, a new theoretical framework must be developed to address these gaps.

### Practical pathways for grassland ecological restoration

**Figure 3:**
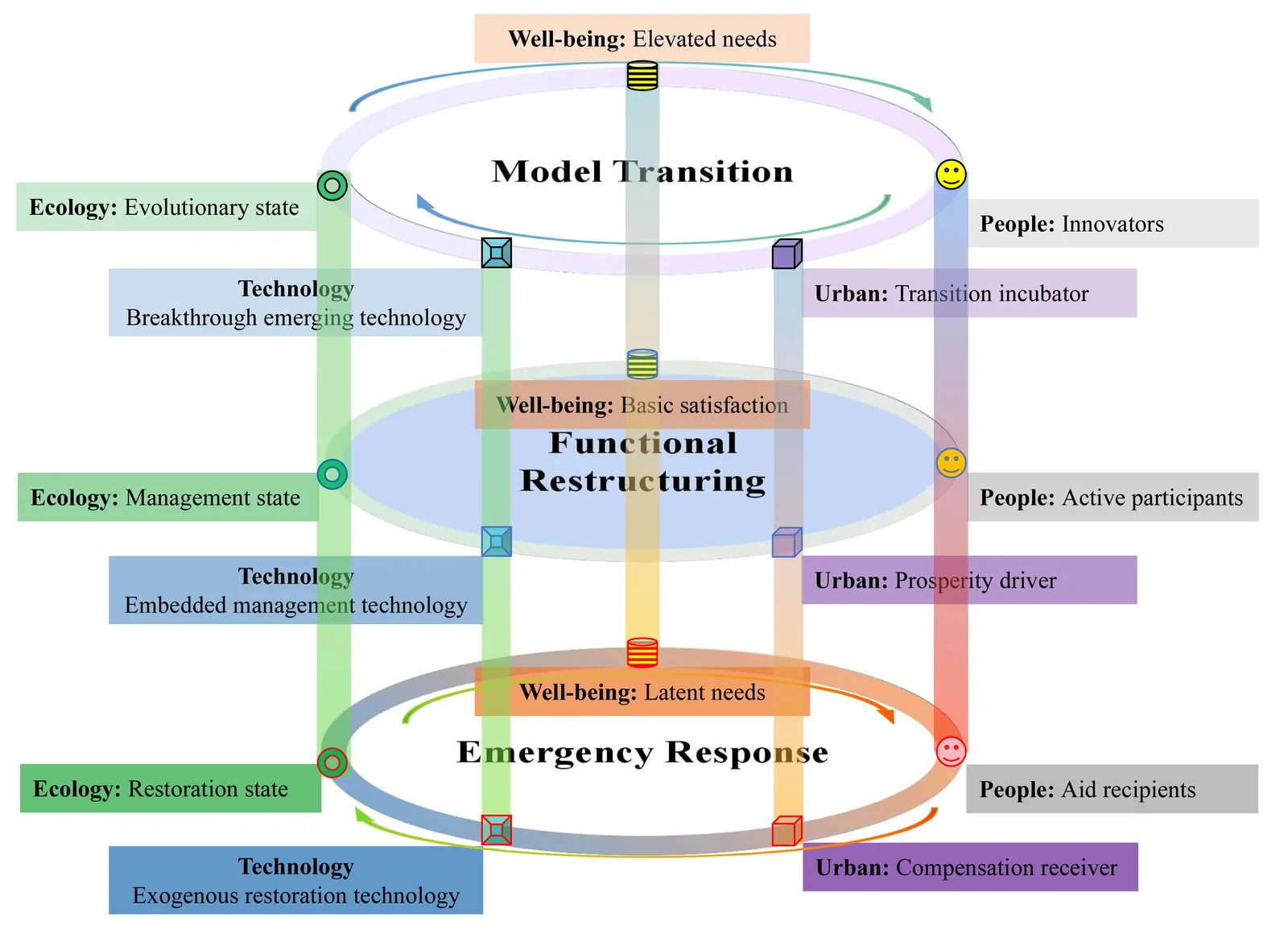
The T-E-W-P-C-T conceptual cycle. The model illustrates the proposed virtuous cycle initiated by transferring exogenous glycine betaine (GB) technology from agricultural stress tolerance to grassland ecological restoration. The five nodes—Technology, Ecology, Well-being, People, and City—are connected in a unidirectional loop (Technology → Ecology → Well-being *rightarrow* People → City → Technology). The model redefines GB as a dynamically evolving agent within the social-ecological system and outlines a three-phase evolutionary pathway (Emergency Response, Functional Restructuring, Model Transition). For detailed phase characteristics, see Tables S3–S6.

Current research on grassland ecological governance generally adopts a systemic perspective. However, it often treats technology as a static tool. It overlooks technology’s dynamic role and evolving contribution within the social-economic-ecological complex. The bibliometric analysis has revealed a clear gap in exogenous GB research applied to grassland ecological restoration. The studies on keystone native species, field validation, and ecosystem-level assessments are largely missing. Building on this gap, this section adopts a “technology-ecology-society” integrated lens. It proposes a conceptual five-node cyclic model to address the question of what social-ecological values might emerge beyond scientific discovery alone if GB technology is transferred from agriculture to grassland restoration (Fig. 3).

#### Logical starting point for model design

Three core findings from the bibliometric analysis constitute the logical starting point for model design. First, the near-absence of studies on keystone native grassland species indicates that technology transfer must begin with high-intensity external intervention—hence Phase I: Emergency Response. Second, the lack of ecosystem-level assessments suggests that once ecological conditions stabilize, the research focus must shift from “restoration” to “management”—forming the core proposition of Phase II: Functional Restructuring. Third, the complementary yet insufficiently integrated patterns of international mechanism-deepening and domestic application-expanding research show that the emergence of new demands drives technological iteration and system transition. This design responds to current research inadequacies and points toward higher-level breakthroughs.

The five nodes of the model are also grounded in the bibliometric findings. The technology-ecology interaction extends the core relationship in agricultural research from cultivated species to keystone grassland species. The introduction of “well-being”, “people”, and “cities” addresses the systemic neglect of the social-ecological dimension. The societal need is inferred from research gaps, in which farmers lack economic value and urban residents lack ecological value.

#### Model structure and core nodes

This model consists of five core nodes, forming a closed loop through unidirectional pathways of the “Technology → Ecology → Well-being → People → City → Technology” cycle (T-E-W-P-C-T cycle). The positioning and connotations of each node are as follows (Table S3):

Technology: The initial driving force that initiates the cycle. In this study, it specifically refers to exogenous GB technology and its application in grassland restoration.

Ecology: The natural foundation upon which human survival depends. In this study, it refers to grassland ecosystems, serving as the source of value generation.

Well-being: The satisfaction of urban and rural residents with their quality of life, encompassing material well-being and spiritual fulfillment. It is the ultimate goal of the model.

People: The foundation of social operation, encompassing human resources at all levels from grassroots to high-end (including rural labor and urban professionals). They are both creators of value and experiencers of well-being.

City: The comprehensive effect generated by population agglomeration, reflected in urban prosperity. It serves as the spatial carrier of human agglomeration and social and economic development.

#### Three-phase evolutionary pathway

Over time, the model evolves driven by societal needs (the “traction force”) and the cycle’s dynamics (the “driving force”). These are closely tied to the phase of evolution. The core characteristics and transmission mechanisms of the three phases are summarized in Tables S3 and S4. The observable indicators and testable pathways are presented in Tables S5 and S6 . The detailed pathways are as follows (Fig. 3).

Phase I (Emergency Response). The “Technology → Ecology” pathway is the core of the cycle. Exogenous GB acts as the initiating factor, which is applied to halt ecosystem degradation. Ecology functions as the primary dependent variable, shifting from a degraded state to a restorative mode. Passively receiving technological inputs, it enables vegetation recovery, soil improvement, and reconstruction of basic ecosystem functions. However, it has not yet translated into tangible benefits for residents. When ecological conditions stabilize, the diminishing marginal benefits of the technology steer the system toward management optimization, triggering Phase II (Table S4).

Phase II (Functional Restructuring). The “Ecology → Well-being” pathway becomes the new core of the cycle. The dominant role of technology shifts toward synergy, focusing on precision enhancement (monitoring, early warning, intervention) to serve ecological management. Ecology becomes the dominant factor in this phase. It shifts from restorative to sustained management. This is embodied in the management of ecosystem service values. As the core of value management, ecology provides rural and urban residents with the economic and ecological values they previously lacked, improving their well-being. Technology transitions from a dominant to a supportive role. It ultimately becomes internalized as an institutional element. It is embedded in the institutional framework of ecological management and evolves into standards, norms, and cultural consensus (Table S4).

Phase III (Model Transition). Ecological management becomes institutionalized. Ecosystem service values reach the current upper limit of the model. At this point, the satisfaction of existing demands gives rise to new demands, and the system enters a phase of free development and innovation driven by “Well-being → People”: urban and rural residents develop higher-level needs, guiding human innovation directions. Subsequently, the “People → City” pathway emerges: new demands drive urban industrial diversification and scaling, leading to the reallocation of resources. Finally, the “City → Technology” pathway unfolds: the demands and resources generated by urban prosperity select “new technologies” capable of addressing emerging social contradictions. This drives the model to transition to a higher level. Overall, this phase tends toward dynamic equilibrium. Internal evolution is rapid, and demand for future technologies is strong. For instance, GB-enhanced stress tolerance could become a criterion for grass seed certification standards. Alternatively, GB application protocols could be incorporated into ecological compensation program guidelines (Table S4).

#### Model nature and research value

This model is a conceptual model. It explicitly proposes assumptions for future research. These include the transmission mechanisms at each node, the pathways for value realization at each phase, and how new demands emerge and drive system transition. The core value of the model lies in articulating that the transfer of GB technology from agriculture to grassland restoration could initiate a virtuous cycle. In this cycle, rural and urban residents respectively gain the economic and ecological values they currently lack. Well-being drives the stabilization and agglomeration of human resources. Urban prosperity reinforces technological innovation. The satisfaction of existing demands generates new demands.

In addition, this model provides a common foundation and distinct perspectives for policy formulation, project design, and interdisciplinary dialogue. It also offers a transferable conceptual model for the migration of other agricultural technologies into ecological restoration domains.

## Discussion

### Research positioning

The core findings of this study can be understood at two levels. At the empirical level, bibliometric analysis reveals two characteristics of exogenous GB research. First, international and domestic studies exhibit a complementary pattern of “mechanism-deepening” *versus* “application-expanding”. Second, a significant structural gap exists in the translation to grassland ecological restoration (Lyu et al., 2024; Ding et al., 2023). Research on keystone native species, field validation, and ecosystem-level assessments is rarely addressed. This finding, derived from keyword co-occurrence clustering (Fig. 2 & Fig. S1) and institutional collaboration patterns (Fig. S2), corroborates the disconnection between high-efficiency restoration technologies and social-ecological systems realities in the context of global grassland degradation (Huang & Hou, 2024; Assan et al., 2025).

At the theoretical level, this gap reflects a paradigm misalignment between agricultural research and ecological restoration: the former follows the linear logic of “crop individual → yield”. The latter demands multi-scale integration across “species → community → ecosystem”. Building on this, the five-node cyclic model proposed as the T-E-W-P-C-T cycle situates technological intervention within the dynamic evolution of social-ecological systems. It reveals the evolutionary logic of technology from external intervention to institutional internalization and ultimately to system transition. This transcends the linear paradigm of traditional technology diffusion theory (Silberg et al., 2024).

Compared with existing research, the contribution of this model lies in three dimensions of expansion. In terms of analytical framework, existing grassland restoration studies have largely focused on technology effect assessment or ecological process simulation (Wan et al., 2026; Temperton et al., 2025). By incorporating “well-being”, “people”, and “city” into the framework, this model directly links technological application with social welfare. It responds to scholarly calls for “technology-society collaborative governance” (Li, Taeihagh & Tan, 2022; Nakano & Washizu, 2021). It shifts ecological restoration research from a singular natural science paradigm toward an integrated social-ecological systems perspective (Khamassi et al., 2026; Yu et al., 2025). In terms of temporal dimension, the strength of social-ecological systems theory lies in identifying structural elements. Yet it pays insufficient attention to role transformation over time (Khodaparast et al., 2025). Through its three-stage division, this model concretizes the evolutionary process of technology from “external intervention” to “institutional internalization” and then to “system self-drive”. It provides a temporal analytical tool for understanding the long-term evolution of technology within social-ecological systems (van Riper et al., 2018). In terms of evolutionary mechanism, traditional technology diffusion theory focuses on the one-way process from R&D to application (Wang, Xu & Wang, 2025). It overlooks how the adaptive responses of social-ecological systems after technology adoption can reciprocally shape technological development pathways. Through the feedback loop of “City → Technology”, this model elucidates how social demands drive technological iteration. It incorporates the “second-order effects” of technology adoption into the analytical framework. This offers a new analytical tool for understanding the co-evolutionary relationship between technology and society.

### Mechanism analysis

Furthermore, the intrinsic logic of this model can be summarized in terms of three core mechanisms. The value transformation mechanism reveals the stepwise pattern of technological value conversion (Xu et al., 2025; Dasgupta & Shakya, 2023). Material inputs are transformed into ecological outputs, then into social welfare, and ultimately back to human capital (McBain, 2015). This explains why technological intervention can generate social value that extends beyond ecological benefits. The actor activation mechanism explains the evolving role of “people” within the model (Koch, Gorris & Pahl-Wostl, 2021). They shift from passive recipients to active participants and then to innovation leaders. This reflects that the fundamental goal of technological intervention is not to permanently replace the functions of actors, but to activate endogenous agency through value creation (Lee, Lee & He, 2025). The system feedback mechanism reveals the dual role of the city within the social-ecological system (Zhang et al., 2025). It serves both as a beneficiary of value and an incubator of innovation. Through the “City → Technology” feedback loop, social demands drive technological iteration, forming a cycle of continuous evolution (Donges et al., 2021). The underlying rationale for the three-stage division lies in the depth of coupling between technology and the social-ecological system. It progresses from a “technology-dominated” phase (Phase I) to a “technology-system synergy” (Phase II) phase and finally to a “system self-organization” phase (Phase III). This evolutionary logic reveals the fundamental pattern of transferring agricultural technologies to ecological restoration: the ultimate goal of technology is not permanent presence, but rather to cultivate the system’s own self-sustaining capacity.

### Limitations and challenges

The limitations of this study can be understood from three perspectives, which reflect not only the inherent characteristics of conceptual models in the theoretical construction phase but also the specific features of the proposed model.

The lack of empirical validation is a core limitation of this study. The five-node model and its three-stage evolutionary pathway remain at the hypothesis stage. Their validity and effectiveness await empirical testing. This limitation stems from the methodological orientation of this study (Lyu, Liu & Yao, 2023; Gaviria-Marin, Merigó & Baier-Fuentes, 2019). Bibliometric analysis can identify research gaps but cannot directly validate model assumptions (Dar, Badwan & Kumar, 2024; Victorino & Peña, 2023). To address this, the study has systematically outlined observable indicators and testable pathways for each node relationship across the stages in the appendix (Table S5). This provides an operational framework for future empirical research.

At the same time, the temporal scale and triggering conditions for the three-stage evolution remain unclear, and more research support is needed (Table S6). Although the model describes the progressive relationships between stages, it does not specify the thresholds for transitioning from Phase I to Phase II. Examples include the level of vegetation cover recovery or the degree of community participation required (Crowe et al., 2013). This limitation reflects the nonlinear nature of the social-ecological system evolution (Tedesco et al., 2023). Lag effects exist between ecological restoration and social responses (Da Silva et al., 2025). Triggering conditions often vary by context. This makes it challenging to translate the model into actionable decision-making tools.

Moreover, the application of the model faces multiple practical challenges. The application of GB may increase production costs, affecting herders’ willingness to adopt it (Taskin & Ertan, 2023; Musajan et al., 2024; Yang et al., 2022a; Yang et al., 2022b). New technologies must be deeply integrated with measures such as grazing management and grassland improvement (Ali & Kaul, 2025). This imposes higher demands on the grassland technology extension system. Converting long-term ecological value into short-term benefits for herders requires precise management strategies and policy incentives (Taskin & Ertan, 2023; Yang et al., 2022a; Yang et al., 2022b; Yi et al., 2020). These three challenges are rooted in the “value time lag” inherent in the promotion of ecological restoration technologies (Deng et al., 2025). Ecological benefits accrue over the long term, while costs are incurred in the short term (Golub et al., 2021; Sun et al., 2021). This temporal mismatch represents a structural challenge common to the application of such technologies.

### Research prospects

Building on the above limitations, future research should proceed along three key areas: empirical validation, model optimization, and application exploration.

In terms of empirical validation, systematic testing of the model’s hypotheses is required. First, field-based observational studies should be strengthened. Technologies such as remote sensing, GIS, and unmanned aerial vehicles should be integrated (Ishikawa, Kyttä & Rinne, 2026; Liu et al., 2022; Soubry et al., 2021; Perevochtchikova & Negrete, 2015). Physiological effect experiments of GB on keystone grassland species should be conducted. Multi-scale field monitoring networks should be established to assess ecosystem-level responses, including community structure, soil health, and carbon sequestration functions (Tian et al., 2017). Second, social surveys should be conducted. Changes in the well-being of urban and rural residents and their behavioral impacts should be tracked (Guo, Zeng & Xia, 2025). The influence of economic and ecological values on satisfaction levels should be quantified (Tang et al., 2025; Ziegler, 2021). This will provide empirical evidence for the “Well-being → People” transmission mechanism.

In terms of model optimization, the triggering conditions and temporal scales of the three-stage evolution need to be clarified. Adaptive management frameworks can serve as a reference (Kimball & Lulow, 2019). Comparative case studies can be used to identify key thresholds for the transition from Phase I to Phase II. These thresholds should be parameterized to enhance the model’s practical applicability (Güler & Büyükkaya, 2024). Meanwhile, mechanisms for mitigating the “value time lag” should be explored (Li et al., 2020). Specifically, how policy tools such as ecological compensation and carbon trading can convert long-term ecological value into short-term economic benefits should be investigated (Xiong et al., 2025; Fu et al., 2025; Xian et al., 2024). This will help overcome the adoption barriers associated with technology dissemination.

In terms of application exploration, collaborative “government-research-enterprise” translation models can be pursued through small-scale pilot projects to test technological suitability and community acceptance (Kwon, 2024; Goodarzi et al., 2021). A risk monitoring and feedback mechanism for technology application should also be established (Cheng et al., 2025). This will ensure that the model possesses self-correcting capacity during empirical testing. At the same time, attention should be paid to institutional innovation in the technology dissemination process (Li & Deng, 2025). Specifically, how GB technology can be internalized as an institutional element of grassland management rather than relying on long-term external intervention.

In summary, the five-node cyclic model proposed in this study serves as a heuristic tool rather than a practical operational guide. Its value lies in providing a testable theoretical framework. It systematically presents the possible pathways and potential values associated with transferring GB technology from agriculture to grassland restoration. Future research can follow the above pathways to test the model’s validity. This will provide empirical support for the effective translation of agricultural technologies into the field of ecological restoration.

## Conclusion

This study systematically reviews exogenous GB research. It has developed a substantial knowledge base and technical accumulation in the field of agricultural stress tolerance. However, its migration to grassland ecological restoration is hindered by a near absence of studies on keystone native grassland species, field validation, and ecosystem-level assessments. International and domestic research exhibit a complementary pattern of “mechanism-deepening” and “application-expanding” trajectories. Yet they remain insufficiently integrated in addressing combined stresses and ecosystem-scale integration.

Based on this identified research gap, this study adopted a “technology-ecology-society” integrated perspective. It proposed the conceptual T-E-W-P-C-T cycle and outlined a three-phase evolutionary pathway: Phase I (Emergency Response), Phase II (Functional Restructuring), and Phase III (Model Transition). The core value of this model lies in elucidating how the transfer of GB technology from agriculture to grassland restoration could initiate a virtuous cycle. In this cycle, urban and rural residents respectively obtain the economic and ecological values they currently lack.

Methodologically, this study combines bibliometric analysis with conceptual model construction. It offers a replicable methodological framework for cross-domain application of agricultural technologies. Theoretically, it redefines GB from a static “technological tool” to a dynamically evolving role within social-ecological systems. It expands the application boundaries of technology diffusion theory. Practically, it specifies the transmission mechanisms at each node and the pathways for value realization at each stage. It provides a theoretical foundation for policy formulation and interdisciplinary dialogue.

The model proposed in this study is a heuristic thinking tool rather than a practical operational guide. Future research should conduct targeted empirical validation of the model’s hypotheses. This includes verifying GB’s physiological effects on keystone grassland species, conducting multi-scale field experiments to assess ecosystem-level responses, tracking changes in urban and rural residents’ well-being and their behavioral impacts through social surveys, and exploring governance models integrating “data-driven approaches, community participation, and policy coordination”. These studies will test the validity of the model. They will provide empirical support for the effective translation of GB technology from agricultural to grassland ecological restoration.

## Supplemental Information

10.7717/peerj.21523/supp-1 Supplemental Information 1 Supplemental tables and figures

10.7717/peerj.21523/supp-2 Supplemental Information 2 PRISMA checklist

10.7717/peerj.21523/supp-3 Supplemental Information 3 The full list of the 295 screened core publications

10.7717/peerj.21523/supp-4 Supplemental Information 4 Data Worksheets The core column names used in the worksheets record publication or occurrence counts (weight, weight), total citations and normalized citations (weight, weight), as well as average publication year, average citations, and average normalized citations (score, score, score). Network and clustering information is provided by cluster ID (cluster), number of links (weight), and total link strength (weight). In the keyword-specific sheets, additional measures such as degree (Degree), betweenness centrality (Centrality), PageRank, Sigma, and burst indicators (Burst, BurstBegin , BurstEnd) are included. Node identification is given by labels (label), full literature descriptions (description), DOI links (url), and visualisation coordinates (x, y). Some fields (*e.g.,* Half-life, Vol, Page) are reserved but remain empty.

10.7717/peerj.21523/supp-5 Supplemental Information 5 295 research articles, with titles highly focused on the application of exogenous glycine betaine (GB) in plant stress physiology Exogenous glycine betaine (GB) alleviates a wide range of abiotic stresses (such as salinity, drought, extreme temperatures, and heavy metal toxicity) through multiple coordinated mechanisms. It reduces oxidative damage by increasing the activities of antioxidant enzymes (SOD, POD, CAT) and decreasing the levels of MDA and reactive oxygen species. GB also helps maintain osmotic balance by promoting the accumulation of proline, soluble sugars, and proteins, thereby improving plant water status. In addition, it protects the photosynthetic apparatus by raising chlorophyll content, net photosynthetic rate, stomatal conductance, and chlorophyll fluorescence parameters (Fv/Fm), while alleviating photoinhibition. GB regulates ion homeostasis by reducing Na^+^ and heavy metal uptake and enhancing K^+^ accumulation. These physiological changes lead to improved seed germination, biomass production, root and shoot growth, and fruit quality. Moreover, GB upregulates stress-related genes, including those encoding antioxidant enzymes, heat shock proteins, and GB biosynthetic enzymes. The optimal concentration of GB (typically 1–100 mM) and application method (seed soaking, root irrigation, or foliar spray) vary with crop species and stress type, but GB is particularly effective in plants that do not naturally accumulate it and often acts synergistically with proline, salicylic acid, or calcium chloride. Overall, exogenous GB is an efficient and low-cost protectant for enhancing crop stress tolerance and productivity.

## Additional Information and Declarations

### Data Availability

The following information was supplied regarding data availability: This is a Literature Review.
