# Supplementary material for: Constructing pathways for glycine betaine in cross-domain ecological restoration: a bibliometric analysis
Source: PeerJ. 2026 Aug 21;14:e21523. doi: 10.7717/peerj.21523 (PMC13505525; doi:10.7717/peerj.21523)
Supplement: Supplementary file 1 [file peerj-14-21523-s001.docx]

**Table S1. Glycine betaine application concentrations for major study subjects**

| **Study Subject** | **Cultivar/Variety** | **Optimal Concentration** | **Application Method** | **Reference Source** |
| --- | --- | --- | --- | --- |
| Rice | Zhonghua 11 | 5-10 mmol/L | Root Application | Zhao et al., Acta Agriculturae Boreali-Sinica, 2006 |
| Buckwheat | Buckwheat TQ-0808 | 60 mmol/L | Root Application | Yang Hongbing, Guizhou Agricultural Sciences, 2013 |
| Maize | Hanyu 5 and Taidan 32 | 0.5 mmol/L | Foliar Spray | Huang Yichun et al., Journal of Maize Sciences, 2011 |
| Soybean | Zhechun 3 | 400 mg/L | Root Application | Huang Yingyun et al., Acta Agriculturae Zhejiangensis, 2012 |
| Tomato | Zhongza 101 | 5 mmol/L | Root Application | Zhou Rong et al., Journal of Anhui Agricultural Sciences, 2008 |
| Apple | Yanfu 3 / M9T337 | 200 mmol/L | Foliar Spray | Ma Mingzhen, South China Fruits, 2016 |
| Pomegranate | Taishan Red Pomegranate | 200 mmol/L | Foliar Spray | Fan Chunli et al., Jiangsu Agricultural Sciences, 2016 |
| Tobacco | K326 | 10-15 mmol/L | Foliar Spray | Qiu Nianwei, Scientia Agricultura Sinica, 2008 |
| Common Glasswort | Common Glasswort | 5 mmol/L | Seed Soaking | Zhou Quancheng et al., Agricultural Research in the Arid Areas, 2009 |
| Seepweed | Seepweed | 20 mmol/L | Seed Soaking | He Lidan et al., Pratacultural Science, 2013 |
| Wheat | Xinong 918, Jinmai 8911 | 25-50 mg/kg | Foliar Spray | Yang Shuying et al., Acta Botanica Boreali-Occidentalia Sinica, 2000; Zhang Jianxin et al., Agricultural Research in the Arid Areas, 2003 |
| Alfalfa | Gannong No.3, Gannong No.5 | 30 mmol/L | Foliar Spray | Lu Xuping et al., Grassland and Turf, 2019; Dong Wenke et al., Acta Agrestia Sinica, 2019 |
| Kentucky Bluegrass | Midnight, Dwarf Mutant A16 | 50 mmol/L | Foliar Spray | Ling Xinwen, Beijing Forestry University, 2020 |
| Creeping Bentgrass | Penn-A4 | 25 mmol/L | Foliar Spray | Zhou Rong, Gansu Agricultural University, 2018 |
| Creeping Bentgrass | T-1 | 100 mmol/L | Foliar Spray | Liu Silu, Beijing Forestry University, 2015 |
| Creeping Bentgrass | Pennncro | 200 mmol/L | Foliar Spray | Liu Nanqing, Nanjing Agricultural University, 2020 |

Note: The table lists representative studies on exogenous glycine betaine (GB) application in various plant species in various plant species. For each study subject, the cultivar/variety, optimal concentration, application method, and reference source are provided. Concentrations are given in mmol/L or mg/L as originally reported. This summary is based on literature retrieved from CNKI (1980–2023).

**Table S2. Co-citation metrics of key researchers in exogenous GB research**

| Author | Papers Number | Centrality | Burst | Burst Begin | Burst End | Half-life |
| --- | --- | --- | --- | --- | --- | --- |
| Allard F | 23 | 0.0417 | 5.2312 | 1998 | 2009 | 7.5 |
| Annunziata MG | 14 | 0.0151 | 5.1240 | 2020 | 2023 | 0.5 |
| Arakawa K | 9 | 0.0232 | 3.9449 | 1992 | 2009 | 7.5 |
| Bates LS | 43 | 0.0657 | 4.1808 | 2021 | 2023 | 32.5 |
| Chen WP | 13 | 0.0416 | 4.1520 | 2004 | 2011 | 1.5 |
| Coughlan SJ | 7 | 0.0008 | 4.3626 | 1996 | 2001 | 2.5 |
| Demiral T | 40 | 0.0149 | 4.4511 | 2011 | 2014 | 6.5 |
| Giri J | 15 | 0.0061 | 3.8646 | 2015 | 2021 | 5.5 |
| Greenway H | 10 | 0.0342 | 4.4166 | 1982 | 2008 | 16.5 |
| Gupta N | 13 | 0.0323 | 3.9670 | 2019 | 2023 | 0.5 |
| Hanson AD | 26 | 0.0721 | 8.3656 | 1987 | 2000 | 12.5 |
| Harinasut P | 22 | 0.0035 | 5.6550 | 2002 | 2012 | 5.5 |
| Hasanuzzaman M | 28 | 0.0022 | 7.8020 | 2018 | 2023 | 2.5 |
| Hayashi H | 13 | 0.0476 | 5.0577 | 1998 | 2008 | 7.5 |
| Holmström KO | 12 | 0.0017 | 6.4359 | 2004 | 2009 | 1.5 |
| Incharoensakdi A | 11 | 0.0342 | 5.2888 | 1988 | 2007 | 14.5 |
| Jolivet Y | 7 | 0.0614 | 4.0817 | 1987 | 2003 | 11.5 |
| Jones RGW | 6 | 0.0173 | 3.7847 | 1997 | 2001 | 0.5 |
| Jones RGW | 8 | 0.0314 | 4.8250 | 1987 | 2001 | 8.5 |
| Kishitani S | 21 | 0.0351 | 5.9423 | 1998 | 2006 | 7.5 |
| Lopez CML | 9 | 0.0098 | 3.9025 | 2006 | 2012 | 2.5 |
| Mccue KF | 18 | 0.0517 | 4.2858 | 1996 | 2001 | 10.5 |
| Murata N | 12 | 0.0470 | 6.2738 | 1996 | 2006 | 4.5 |
| Nomura M | 15 | 0.0088 | 4.4948 | 1999 | 2009 | 6.5 |
| Osman HS | 13 | 0.0119 | 3.9670 | 2019 | 2023 | 0.5 |
| Papageorgiou GC | 20 | 0.0256 | 5.3903 | 1998 | 2006 | 7.5 |
| Rahman S | 16 | 0.0182 | 3.8371 | 2011 | 2018 | 2.5 |
| Rathinasabapathi B | 10 | 0.0384 | 4.6834 | 1998 | 2009 | 7.5 |
| Raza MAS | 14 | 0.0256 | 4.2740 | 2019 | 2023 | 1.5 |
| Rhodes D | 58 | 0.1727 | 3.8842 | 1997 | 2012 | 13.5 |
| Robinson SP | 16 | 0.0247 | 7.5135 | 1998 | 2009 | 5.5 |
| Smirnoff N | 10 | 0.0100 | 4.3381 | 2006 | 2012 | 2.5 |
| Wang GP | 23 | 0.0411 | 3.7882 | 2013 | 2017 | 5.5 |
| Yancey PH | 8 | 0.0675 | 3.8287 | 1997 | 2008 | 8.5 |
| Yang XH | 39 | 0.0541 | 4.7630 | 2011 | 2015 | 7.5 |
| Yildirim E | 17 | 0.0240 | 5.1752 | 2015 | 2021 | 3.5 |
| Zhang LX | 20 | 0.0043 | 4.8913 | 2014 | 2021 | 4.5 |

Note: The table presents bibliometric indicators for researchers with relatively high co-citation frequency in the field of exogenous glycine betaine (GB) research. Metrics include number of papers, centrality, citation burst strength, burst start and end years, and half-life period. Data are derived from the Web of Science Core Collection (1980–2023) and analyzed using CiteSpace.

**Table S3. Core characteristics of the five nodes across the three evolutionary phases**

| ****Node**** | ****Phase I: Emergency Response**** | ****Phase II: Functional Restructuring**** | ****Phase III: Model Transition**** |
| --- | --- | --- | --- |
| Technology | Exogenous restoration technology  (High-intensity restoration technology introduced externally) | Embedded management technology  (Precision management technology integrated into the system) | Breakthrough emerging technology  (Transformative technology responding to new needs) |
| Ecology | Restoration state  (Object of restoration) | Managed state  (Value hub under active management) | Evolutionary state  (Living system co-evolving with human needs) |
| Well-being | Latent needs  (Unmet survival needs) | Basic satisfaction  (Material needs met and enhanced liveability) | Elevated needs  (Emerging needs for spiritual culture and self-actualization) |
| People | Recipients  (Passively receiving assistance) | Participants  (Actively engaging in co-construction) | Innovators  (Leading innovation and reallocating resources) |
| Urban | Compensation receiver  (Receiving ecological compensation) | Prosperity driver  (Driving prosperity through workforce agglomeration) | Transition incubator  (Nurturing new technologies through resource flow) |

Note: The table summarizes the changing roles and states of the five nodes (Technology, Ecology, Well-being, People, Urban) in the T-E-W-P-C-T cycle during Phase I (Emergency Response), Phase II (Functional Restructuring), and Phase III (Model Transition). Each node’s attributes evolve from externally driven to system-embedded and finally to innovation-oriented.

**Table S4. Interaction pathways and practical implications of the five nodes across three phases**

| ****Interaction**** | ****Phase I: Emergency Response**** | ****Phase II: Functional Restructuring**** | ****Phase III: Model Transition**** |
| --- | --- | --- | --- |
| Technology → Ecology | High-intensity input initiates restoration | Smart monitoring, precision intervention | New technology empowers model transition |
| Ecology → Well-being | Ecological recovery, value untransformed | Economic value to farmers, ecological value to citizens | Synergy with new needs, well-being upgraded |
| Well-being → People | Behavioral change not triggered | Rural workforce stabilizes, urban workforce agglomerates | New needs guide innovation, resource reallocation |
| People → Urban | Initial workforce stabilization, urban effects not yet visible | Urban-rural workforce synergy drives prosperity | Innovation-led resource reallocation incubates transition |
| Urban → Technology | One-way compensation, no feedback | Prosperity generates demand, initial feedback | Resource allocation incubates new technology |

Note: The table describes the dominant interactions between consecutive nodes (Technology→Ecology, Ecology→Well-being, Well-being→People, People→Urban, Urban→Technology) in each phase. It highlights the shift from one-way technological input (Phase I) to synergistic management (Phase II) and finally to self-driven innovation and resource reallocation (Phase III).

**Table S5. Observable indicators for testing the model across three phases**

| **Interaction** | **Phase I: Emergency Response** | **Phase II: Functional Restructuring** | **Phase III: Model Transition** |
| --- | --- | --- | --- |
| Technology → Ecology | ****Scientific validation with technological applicability**** • Density and biomass of keystone grassland species • Vegetation coverage, species diversity index • Soil organic matter, electrical conductivity, microbial biomass carbon | ****Technology promotion at scale with effective ecological restoration**** • Area under technology promotion, number of adopting farmers/cooperatives • Stability of vegetation recovery (interannual coefficient of variation) • Ecosystem function recovery index | ****Ecological restoration targets achieved**** • Achievement rate of target species restoration area • Ecosystem self-maintenance capacity • Similarity to reference ecosystem |
| Ecology → Well-being | ****Foundation of ecosystem services**** • Vegetation coverage, species richness • Water conservation capacity, soil retention • Soil organic carbon storage | ****Objective ecosystem services + residents' perceived benefits**** • Air quality index (PM2.5, negative oxygen ions) • Herder household income, livestock production benefits • Urban residents' satisfaction with ecotourism, willingness to pay | ****Full realization of service value**** • Ecosystem service value • Enhancement of carbon sink function • Composite index of urban and rural residents' well-being |
| Well-being → People | — | ****Stabilization of local population**** • Net out-migration rate of rural population • Participation rate of urban and rural residents in ecotourism • Number of returnees starting businesses | ****Agglomeration of talent**** • In-migration rate of external population • Retention rate of highly educated talent • Inflow rate of professional and technical personnel |
| People → Urban | — | ****Incubation of local industries**** • Share of ecotourism income in township GDP  • Number of newly registered local small and micro enterprises  • Proportion of households driven by cooperatives | ****Industrial establishment + urban growth**** • Investment amount and number of projects from external enterprises • Urban industrial diversification index • Urban GDP growth rate, employment rate |
| Urban → Technology | — | ****Initiation of technology investment**** • Local government fiscal investment in grassland restoration technologies • Number of enterprise-participated ecological restoration projects | ****Formation of innovation ecosystem**** • Number of new technology patents granted • Number and funding of industry-university-research collaboration projects • Share of green technology R&D investment in GDP • Intellectual property commercialization rate |

Note: The table provides a set of measurable indicators for each node-to-node interaction in Phases I, II, and III. Indicators cover ecological (vegetation coverage, soil carbon, species diversity), economic (herder income, ecotourism revenue), social (population stability, talent agglomeration), and technological (technology adoption area, innovation investment) dimensions. These indicators are intended for empirical validation of the conceptual model.

**Table S6. Methodological approaches and assessment techniques for model testing across three phases**

| **Interaction** | **Phase I: Emergency Response** | **Phase II: Functional Restructuring** | **Phase III: Model Transition** |
| --- | --- | --- | --- |
| **Technology → Ecology** | ****Scientific validation through experimentation**** Conduct field control experiments with gradient concentrations of glycine betaine; long-term monitoring of physiological and ecological indicators of keystone species | ****Technology promotion assessment**** Quantify application area and coverage of precision management technologies (e.g., drones, sensors); compare ecological restoration outcomes under different management measures | ****Ecological restoration effectiveness evaluation**** Conduct long-term monitoring to assess ecosystem self-maintenance capacity; apply reference ecosystem comparison method to determine whether restoration targets are achieved |
| **Ecology → Well-being** | ****Ecosystem function monitoring**** Establish vegetation quadrats and soil sampling points; calculate vegetation coverage and water conservation capacity using remote sensing data; establish carbon flux monitoring towers | ****Residents' perceived benefits survey**** Track herder income through household surveys; conduct willingness-to-pay surveys for grassland ecosystem services using contingent valuation method (CVM); correlate air quality monitoring data with residents' satisfaction | ****Integrated service value assessment**** Evaluate ecosystem service value; monitor carbon sink function; conduct urban and rural residents' well-being scale surveys (e.g., life satisfaction, well-being index) |
| **Well-being → People** | — | ****Population stability survey**** Conduct household surveys on herders' willingness to stay and out-migration status; calculate net out-migration rate of pastoral areas; interview returnees starting businesses | ****Talent mobility tracking**** Analyze in-migration population data from census; survey employment flows of highly educated talent; evaluate effectiveness of talent attraction policies |
| **People → Urban** | — | ****Local industry statistics**** Calculate ecotourism revenue; compile small business registration data; survey the proportion of households driven by cooperatives | ****Urban economic impact assessment**** Query investment promotion project databases; compare urban economic census data; investigate urban-rural factor flows (capital, technology, talent) |
| **Urban → Technology** | — | ****Technology investment statistics**** Retrieve local government technology budgets; query enterprise project databases from development and reform or environmental protection departments | ****Innovation output analysis**** Search patent databases for glycine betaine-related technologies; count industry-university-research collaboration projects; analyze the share of green technology in corporate R&D investment |

Note: This complementary table outlines specific methodological approaches and assessment criteria for each interaction across the three phases. Examples include field control experiments for Technology→Ecology (Phase I), household surveys for Well-being→People (Phase II), and long-term ecosystem monitoring for Ecology→Well-being (Phase III). The table serves as an operational guide for future empirical studies.

**Figure S1. Keyword co-occurrence network of exogenous GB research**

Note: The network visualizes co-occurrence relationships among high-frequency keywords extracted from the literature on exogenous glycine betaine (GB). Node size reflects keyword frequency, and link thickness indicates co-occurrence strength. Clusters represent major research themes (e.g., stress physiology, antioxidant mechanisms, agricultural applications). Analysis was performed using VOSviewer. CN, keywords of Chinese literature.

**Figure S2. Geographic distribution of research institutions publishing on exogenous GB**

Note: The map shows the spatial distribution of institutions that have contributed to exogenous GB research, based on affiliation data from the Web of Science Core Collection (1980-2023). Marker size is proportional to the number of publications per institution. The visualization was generated using Scimago Graphica.

**Figure S3. Researcher co-citation network**

Note: The network illustrates co-citation relationships among authors in the exogenous GB field. Nodes represent cited authors, and links indicate co-citation frequency. The layout highlights clusters of researchers working on related topics (e.g., osmotic adjustment, antioxidant enzymes, genetic engineering). Analysis was performed using CiteSpace.

**Figure S4. Literature citation network**

Note: The network illustrates the co-citation relationships among the 295 articles included in the bibliometric analysis. Nodes represent individual articles, and links indicate that two articles are cited together by subsequent publications. Node size reflects co-citation frequency, and clusters represent groups of articles that form intellectual bases for research fronts. The analysis was performed using CiteSpace (time slices: 1980–2023, threshold: ≥2 citations per slice).

**Figure S5. Literature coupling network**

Note: The network shows bibliographic coupling among the reference lists of the 295 included articles. Two references are coupled if they appear together in the same reference list. This analysis reveals the structural relationships between the knowledge sources used by the 295 articles. Clusters indicate groups of references that are frequently used together, reflecting shared thematic backgrounds. Analysis performed using VOSviewer with full counting method.

**Figure S6. Reference co-citation network**

Note: The figure presents a cluster view of the co-citation network among the references cited by the 295 core articles. The analysis was performed using VOSviewer. Nodes represent individual references, with node size proportional to the total citation frequency. Links indicate co-citation relationships (two references cited together by subsequent publications). Clusters are identified by the VOSviewer clustering algorithm and are distinguished by different colors. The network reveals the intellectual structure of exogenous GB research, grouping foundational references into thematic clusters such as osmotic adjustment, antioxidant defense, photosynthetic protection, and genetic engineering.
