# Supplementary material for: Constructing pathways for glycine betaine in cross-domain ecological restoration: a bibliometric analysis
Source: PeerJ. 2026 Aug 21;14:e21523. doi: 10.7717/peerj.21523 (PMC13505525; doi:10.7717/peerj.21523)
Supplement: Supplementary file 2 [file peerj-14-21523-s002.docx]

| **Section and Topic** | **Item #** | **Checklist item** | **Location where item is reported** |
| --- | --- | --- | --- |
| **TITLE** | | |  |
| Title | 1 | Identify the report as a systematic review. | Page 1, Title: "Constructing pathways for glycine betaine in cross-domain ecological restoration: a bibliometric analysis" |
| **ABSTRACT** | | |  |
| Abstract | 2 | See the PRISMA 2020 for Abstracts checklist. | Page 1, Abstract: Provides Background, Methods (databases, analysis tools), Results (research landscape, gaps, conceptual model), and Conclusion. |
| **INTRODUCTION** | | |  |
| Rationale | 3 | Describe the rationale for the review in the context of existing knowledge. | Page 3, Introduction: Paragraphs 1-3 describe grassland degradation, the potential of GB, and the knowledge gap in applying GB from agriculture to grassland restoration. |
| Objectives | 4 | Provide an explicit statement of the objective(s) or question(s) the review addresses. | Page 4, Introduction: States two explicit questions: (1) Does a genuine gap exist? (2) What social-ecological values might emerge from filling this gap? |
| **METHODS** | | |  |
| Eligibility criteria | 5 | Specify the inclusion and exclusion criteria for the review and how studies were grouped for the syntheses. | Page 6, Literature Screening: Lists inclusion criteria (exogenous GB effects on plant growth) and exclusion criteria (tissue culture, detached organs, reviews, etc.). |
| Information sources | 6 | Specify all databases, registers, websites, organisations, reference lists and other sources searched or consulted to identify studies. Specify the date when each source was last searched or consulted. | Page 5-6, Data Sources: Specifies CNKI and WoS Core Collection. The search cutoff date is given as December 31, 2023. |
| Search strategy | 7 | Present the full search strategies for all databases, registers and websites, including any filters and limits used. | Page 5-6, Data Sources: Provides the exact search queries for CNKI (e.g., SU%='甜菜碱'AND (SU%='添加'OR SU%='外源')) and WoS (e.g., TS= (betaine) AND TS= (addition OR exogenous) NOT WC=...). |
| Selection process | 8 | Specify the methods used to decide whether a study met the inclusion criteria of the review, including how many reviewers screened each record and each report retrieved, whether they worked independently, and if applicable, details of automation tools used in the process. | Page 6, Literature Screening: Describes screening by reading titles and abstracts, and then full text. Figure S1 (referenced) shows the flow. It does not specify the number of reviewers or if they worked independently. |
| Data collection process | 9 | Specify the methods used to collect data from reports, including how many reviewers collected data from each report, whether they worked independently, any processes for obtaining or confirming data from study investigators, and if applicable, details of automation tools used in the process. | Page 6-7, Data Processing and Analysis: Describes converting CNKI data, manual supplementation of reference info, and using VOSviewer and CiteSpace. Does not specify number of reviewers. |
| Data items | 10a | List and define all outcomes for which data were sought. Specify whether all results that were compatible with each outcome domain in each study were sought (e.g. for all measures, time points, analyses), and if not, the methods used to decide which results to collect. | Page 7, Data Processing and Analysis: Lists data sought: authors, affiliations, titles, journals, publication dates, keywords, and references. |
|  | 10b | List and define all other variables for which data were sought (e.g. participant and intervention characteristics, funding sources). Describe any assumptions made about any missing or unclear information. | Not reported. The review does not extract funding sources or detailed study characteristics beyond bibliographic data. |
| Study risk of bias assessment | 11 | Specify the methods used to assess risk of bias in the included studies, including details of the tool(s) used, how many reviewers assessed each study and whether they worked independently, and if applicable, details of automation tools used in the process. | Not applicable. Bibliometric analysis does not typically assess risk of bias of individual studies in the same way as a clinical systematic review. |
| Effect measures | 12 | Specify for each outcome the effect measure(s) (e.g. risk ratio, mean difference) used in the synthesis or presentation of results. | Not applicable. This is a bibliometric review (keyword co-occurrence, citation analysis), not a meta-analysis of effect sizes. |
| Synthesis methods | 13a | Describe the processes used to decide which studies were eligible for each synthesis (e.g. tabulating the study intervention characteristics and comparing against the planned groups for each synthesis (item #5)). | Page 7, Data Processing and Analysis: Describes using CiteSpace for keyword co-occurrence, clustering, burst detection, and timeline analysis. |
|  | 13b | Describe any methods required to prepare the data for presentation or synthesis, such as handling of missing summary statistics, or data conversions. | Page 6-7, Data Processing and Analysis: Describes converting CNKI to CiteSpace format and manually supplementing reference information. |
|  | 13c | Describe any methods used to tabulate or visually display results of individual studies and syntheses. | Page 7, Data Processing and Analysis: Mentions using SCImago Graphica for visualization. Results section includes Figures 1-3 and Tables 1-4. |
|  | 13d | Describe any methods used to synthesize results and provide a rationale for the choice(s). If meta-analysis was performed, describe the model(s), method(s) to identify the presence and extent of statistical heterogeneity, and software package(s) used. | Page 7, Data Processing and Analysis: Specifies VOSviewer (for networks) and CiteSpace (for keyword evolution, clustering), with parameter settings (e.g., time slices, threshold). Cites van Eck & Waltman (2010) and Li & Chen (2016). |
|  | 13e | Describe any methods used to explore possible causes of heterogeneity among study results (e.g. subgroup analysis, meta-regression). | Not applicable. Bibliometric analysis does not explore statistical heterogeneity. |
|  | 13f | Describe any sensitivity analyses conducted to assess robustness of the synthesized results. | Not reported. No sensitivity analysis is described. |
| Reporting bias assessment | 14 | Describe any methods used to assess risk of bias due to missing results in a synthesis (arising from reporting biases). | Not applicable. |
| Certainty assessment | 15 | Describe any methods used to assess certainty (or confidence) in the body of evidence for an outcome. | Not applicable. |
| **RESULTS** | | |  |
| Study selection | 16a | Describe the results of the search and selection process, from the number of records identified in the search to the number of studies included in the review, ideally using a flow diagram. | Page 6, Literature Screening: Describes initial retrieval (206 CNKI, 632 WoS) and final inclusion (132 CNKI, 163 WoS). Figure 1 is the PRISMA flow diagram. |
|  | 16b | Cite studies that might appear to meet the inclusion criteria, but which were excluded, and explain why they were excluded. | Page 6, Literature Screening & Figure 1: Exclusion reasons are listed (tissue culture, detached organs, reviews, etc.), but no specific excluded studies are cited. |
| Study characteristics | 17 | Cite each included study and present its characteristics. | Not presented as a table. Characteristics are synthesized and presented in the text, Tables 1-4, and Figures. A full list of the 295 studies is not provided due to space. |
| Risk of bias in studies | 18 | Present assessments of risk of bias for each included study. | Not applicable. |
| Results of individual studies | 19 | For all outcomes, present, for each study: (a) summary statistics for each group (where appropriate) and (b) an effect estimate and its precision (e.g. confidence/credible interval), ideally using structured tables or plots. | Not applicable. This is not a meta-analysis. |
| Results of syntheses | 20a | For each synthesis, briefly summarise the characteristics and risk of bias among contributing studies. | Page 7-11, Results: Summarizes the characteristics of the research landscape, keyword evolution (Tables 1-2), collaboration networks (Table 3), and knowledge foundation (Table 4). |
|  | 20b | Present results of all statistical syntheses conducted. If meta-analysis was done, present for each the summary estimate and its precision (e.g. confidence/credible interval) and measures of statistical heterogeneity. If comparing groups, describe the direction of the effect. | Page 8-9, Results: Presents results of keyword clustering (Figure 2) and collaboration networks. No meta-analysis was conducted. |
|  | 20c | Present results of all investigations of possible causes of heterogeneity among study results. | Not applicable. |
|  | 20d | Present results of all sensitivity analyses conducted to assess the robustness of the synthesized results. | Not applicable. |
| Reporting biases | 21 | Present assessments of risk of bias due to missing results (arising from reporting biases) for each synthesis assessed. | Not applicable. |
| Certainty of evidence | 22 | Present assessments of certainty (or confidence) in the body of evidence for each outcome assessed. | Not applicable. |
| **DISCUSSION** | | |  |
| Discussion | 23a | Provide a general interpretation of the results in the context of other evidence. | Page 13, Discussion (Research Positioning): Interprets the findings, comparing the "mechanism-deepening" vs. "application-expanding" trajectories with existing literature. |
|  | 23b | Discuss any limitations of the evidence included in the review. | Page 15, Limitations and Challenges: Discusses the lack of empirical validation, unclear temporal scales, and practical challenges of applying GB. |
|  | 23c | Discuss any limitations of the review processes used. | Page 15, Limitations and Challenges: Notes that the conceptual model is a hypothesis, and the lack of empirical validation is a core limitation stemming from the methodological orientation (bibliometric analysis). |
|  | 23d | Discuss implications of the results for practice, policy, and future research. | Page 16, Research Prospects: Outlines future research needs (empirical validation, model optimization). Page 17, Conclusion: Summarizes practical value for policy and interdisciplinary dialogue. |
| **OTHER INFORMATION** | | |  |
| Registration and protocol | 24a | Provide registration information for the review, including register name and registration number, or state that the review was not registered. | Not reported. The review was likely not registered. |
|  | 24b | Indicate where the review protocol can be accessed, or state that a protocol was not prepared. | Not reported. A protocol was likely not prepared. |
|  | 24c | Describe and explain any amendments to information provided at registration or in the protocol. | Not applicable. |
| Support | 25 | Describe sources of financial or non-financial support for the review, and the role of the funders or sponsors in the review. | Not reported. The acknowledgements thank individuals for technical support but do not mention funding sources. |
| Competing interests | 26 | Declare any competing interests of review authors. | Not reported. No competing interests statement is provided. |
| Availability of data, code and other materials | 27 | Report which of the following are publicly available and where they can be found: template data collection forms; data extracted from included studies; data used for all analyses; analytic code; any other materials used in the review. | Not reported. The manuscript does not state where the raw data or analysis code can be found. |
