# Supplementary material for: Constructing pathways for glycine betaine in cross-domain ecological restoration: a bibliometric analysis
Source: PeerJ. 2026 Aug 21;14:e21523. doi: 10.7717/peerj.21523 (PMC13505525; doi:10.7717/peerj.21523)
Supplement: Supplementary file 3 [file peerj-14-21523-s003.docx]

Contents

[1. WoS literature	1](#_Toc21905)

[2. CNKI literature:	16](#_Toc25360)

1. **WoS literature**

Adak N. 2019. Effects of glycine betaine concentrations on the agronomic characteristics of strawberry grown under deficit irrigation conditions. *Applied Ecology and Environmental Research* 17(2):3753-3767. DOI: 10.15666/aeer/1702_37533767

Agboma PC, Jones M, Peltonensainio P, Rita H, and Pehu E. 1997. Exogenous glycinebetaine enhances grain yield of maize, sorghum and wheat grown under two supplementary watering regimes. *Journal of Agronomy and Crop Science* 178(1):29-37. DOI: 10.1111/j.1439-037X.1997.tb00348.x

Agboma PC, Peltonensainio P, Hinkkanen R, and Pehu E. 1997. Effect of foliar application of glycinebetaine on yield components of drought-stressed tobacco plants. *Experimental Agriculture* 33(3):345-352. DOI: 10.1017/S0014479797003062

Ahmad S, Raza I, Ali H, Shahzad AN, Atiq-Ur-Rehman, and Sarwar N. 2014. Response of cotton crop to exogenous application of glycinebetaine under sufficient and scarce water conditions. *Brazilian Journal of Botany* 37(4):407-415. DOI: 10.1007/s40415-014-0092-z

Ahmed N, Zhang Y, Li K, Zhou Y, Zhang M, and Li Z. 2019. Exogenous application of glycine betaine improved water use efficiency in winter wheat (*Triticum aestivum* L.) Via modulating photosynthetic efficiency and antioxidative capacity under conventional and limited irrigation conditions. *The Crop Journal* 7(5):635-650. DOI: 10.1016/j.cj.2019.03.004

Ahmed N, Zhu M, Li Q, Wang X, Wan J, and Zhang Y. 2021. Glycine betaine-mediated root priming improves water stress tolerance in wheat (*Triticum aestivum* L.). *Agriculture* 11(11):1127. DOI: 10.3390/agriculture11111127

Alhoshan M, Zahedi M, Ramin AA, and Sabzalian MR. 2018. Exogenous application of salicylic acid and glycine betaine as tools to enhance biomass and tolerance of potato cultivars. *Gesunde Pflanzen* 71(1):25-35. DOI: 10.1007/s10343-018-00438-2

Ali Q, and Ashraf M. 2011. Exogenously applied glycinebetaine enhances seed and seed oil quality of maize (*Zea mays* L.) Under water deficit conditions. *Environmental and Experimental Botany* 71(2):249-259. DOI: 10.1016/j.envexpbot.2010.12.009

Ali S, Ali H, and Hussain S. 2023. Influence of osmoprotectants and their different application methods on wheat (*Triticum aestivum* L.) Cultivars under water deficiency in south punjab region of pakistan. *Appl. Ecol. Env. Res.* 21(5):3895-3910. DOI: 10.15666/aeer/2105_38953910

Allard F, Houde M, Krol M, Ivanov A, Huner NPA, and Sarhan F. 1998. Betaine improves freezing tolerance in wheat. *Plant and Cell Physiology* 39(11):1194-1202. DOI: 10.1093/oxfordjournals.pcp.a029320

Aminifard MH, Jorkesh A, and Fallahi HR. 2019. Biochemical, morphological and reproductive growth response of fenugreek to foliar application of glycine betaine and salicylic acid. *Zeitschrift Fur Arznei- & Gewurzpflanzen* 24(1):43-48.

Anwar-Ul-Haq M, Iftikhar I, Akhtar J, and Maqsood M. 2023. Role of exogenous osmolyte supplementation in ameliorating osmotic and oxidative stress and promoting growth in salinity-stressed soybean genotypes. *Journal of Soil Science and Plant Nutrition* 23(3):3682-3694. DOI: 10.1007/s42729-023-01289-1

Arakawa K, Mizuno K, Kishitani S, and Takabe T. 1992. Immunological studies of betaine aldehyde dehydrogenase in barley. *Plant and Cell Physiology* 33(7):833-840. DOI: 10.1093/oxfordjournals.pcp.a078331

Aziz M, Ashraf M, and Javaid MM. 2018. Enhancement in cotton growth and yield using novel growth promoting substances under water limited conditions. *Pakistan Journal of Botany* 50(5):1691-1701. https://www.pakbs.org/pjbot/papers/1527614206.pdf

Bharwana SA, Ali S, Farooq MA, Iqbal N, Hameed A, Abbas F, and Ahmad MSA. 2014. Glycine betaine-induced lead toxicity tolerance related to elevated photosynthesis, antioxidant enzymes suppressed lead uptake and oxidative stress in cotton. *Turkish Journal of Botany* 38(2):281-292. DOI: 10.3906/bot-1304-65

Castro-Duque NE, Chávez-Arias CC, and Restrepo-Díaz H. 2020. Foliar glycine betaine or hydrogen peroxide sprays ameliorate waterlogging stress in cape gooseberry. *Plants-Basel* 9(5):644. DOI: 10.3390/plants9050644

Cha Um S, Supaibulwatana K, and Kirdmanee C. 2006. Water relation, photosynthetic ability and growth of Thai Jasmine rice (O*ryza sativa* L. ssp.*indica* cv. KDML 105) to salt stress by application of exogenous glycinebetaine and choline. *J Agronomy Crop Science* 192(1):25-36. DOI: 10.1111/j.1439-037X.2006.00186.x

Chen WP, Li PH, and Chen THH. 2000. Glycinebetaine increases chilling tolerance and reduces chilling‐induced lipid peroxidation in *Zea mays* l. *Plant Cell & Environment* 23(6):609-618. DOI: 10.1046/j.1365-3040.2000.00570.x

Chungloo D, Tisarum R, Samphumphuang T, Sotesaritkul T, Singh HP, Takabe T, and Cha-Um S. 2022. Mitigation of water-deficit stress, physio-morphological adaptation, and elevation of andrographolide in *Andrographis paniculata* using foliar glycine betaine. *Journal of Plant Growth Regulation* 42(10):6273-6285. DOI: 10.1007/s00344-022-10818-8

Cirillo C, Rouphael Y, Caputo R, Raimondi G, Sifola MI, and De Pascale S. 2016. Effects of high salinity and the exogenous application of an osmolyte on growth, photosynthesis, and mineral composition in two ornamental shrubs. *The Journal of Horticultural Science and Biotechnology* 91(1):14-22. DOI: 10.1080/14620316.2015.1110988

Cisse EHM, Li DD, Zhang J, Guo LY, Miao LF, and Yang F. 2022. Responses of woody plant *Dalbergia odorifera* treated with glycine betaine to drought and cold stresses: involvement of the alternative oxidase. *Biologia Plantarum* 6656-66. DOI: 10.32615/bp.2021.062

Cisse EHM, Miao L, Yang F, Huang J, Li D, and Zhang J. 2021. Gly betaine surpasses melatonin to improve salt tolerance in *Dalbergia odorifera*. *Frontiers in Plant Science* 12588847. DOI: 10.3389/fpls.2021.588847

Correia S, Aires A, Queirós F, Carvalho R, Schouten R, Silva AP, and Gonçalves B. 2020. Climate conditions and spray treatments induce shifts in health promoting compounds in cherry (*Prunus avium* L.) fruits. *Scientia Horticulturae* 263109147. DOI: 10.1016/j.scienta.2019.109147

Correia S, Queirós F, Ferreira H, Morais MC, Afonso S, Silva AP, and Gonçalves B. 2020. Foliar application of calcium and growth regulators modulate sweet cherry (*Prunus avium* L.) Tree performance. *Plants-Basel* 9(4):410. DOI: 10.3390/plants9040410

Cruz FJR, Castro GLS, Júnior DDS, Festucci-Buselli RA, and Pinheiro HA. 2013. Exogenous glycine betaine modulates ascorbate peroxidase and catalase activities and prevent lipid peroxidation in mild water-stressed *Carapa guianensis* plants. *Photosynthetica* 51(1):102-108. DOI: 10.1007/s11099-013-0004-7

de Oliveira Maia Júnior S, de Andrade JR, Santos CMD, Santos JV, Silva LKDS, Clemente PRA, Ferreira VM, Silva JV, and Endres L. 2020. Foliar-applied glycine betaine minimizes drought stress-related impact to gas exchange and the photochemical efficiency of PSii in sugarcane. *Theoretical and Experimental Plant Physiology* 32(4):315-329. DOI: 10.1007/s40626-020-00188-5

Demiral T, and Türkan I. 2006. Exogenous glycinebetaine affects growth and proline accumulation and retards senescence in two rice cultivars under nacl stress. *Environmental and Experimental Botany* 56(1):72-79. DOI: 10.1016/j.envexpbot.2005.01.005

Denaxa N, Roussos PA, Damvakaris T, and Stournaras V. 2012. Comparative effects of exogenous glycine betaine, kaolin clay particles and ambiol on photosynthesis, leaf sclerophylly indexes and heat load of olive cv. Chondrolia chalkidikis under drought. *Scientia Horticulturae* 13787-94. DOI: 10.1016/j.scienta.2012.01.012

Díaz-Zorita M, Fernández-Canigia MV, and Grosso GA. 2001. Applications of foliar fertilizers containing glycinebetaine improve wheat yields. *Journal of Agronomy and Crop Science* 186(3):209-215. DOI: 10.1046/j.1439-037X.2001.00469.x

Dima S, Neamțu C, Desliu-Avram M, Ghiurea M, Capra L, Radu E, Stoica R, Faraon V, Zamfiropol-Cristea V, Constantinescu-Aruxandei D, and Oancea F. 2020. Plant biostimulant effects of baker’s yeast vinasse and selenium on tomatoes through foliar fertilization. *Agronomy* 10(1):133. DOI: 10.3390/agronomy10010133

Dong X, Liu Y, Ma X, Wang S, Yang H, Gao X, Wang G, and Wang H. 2024. Disclosing the effect of exogenous betaine on growth of *Suaeda salsa* (L.) Pall in the Liaohe coastal wetland, North China. *Marine Pollution Bulletin* 198115852. DOI: 10.1016/j.marpolbul.2023.115852

Duman F. 2011. Effects of exogenous glycinebetaine and trehalose on lead accumulation in an aquatic plant (*Lemna gibba* L.). *International Journal of Phytoremediation* 13(5):492-497. DOI: 10.1080/15226514.2010.495149

Dustgeer Z, Seleiman MF, Khan I, Chattha MU, Ali EF, Alhammad BA, Jalal RS, Refay Y, and Hassan MU. 2021. Glycine-betaine induced salinity tolerance in maize by regulating the physiological attributes, antioxidant defense system and ionic homeostasis. *Notulae Botanicae Horti Agrobotanici Cluj-Napoca* 49(1):12248. DOI: 10.15835/nbha49112248

Einset J, Winge P, Bones AM, and Connolly EL. 2008. The FRO2 ferric reductase is required for glycine betaine’s effect on chilling tolerance in *Arabidopsis* roots. *Physiologia Plantarum* 134(2):334-341. DOI: 10.1111/j.1399-3054.2008.01141.x

Eisakhani MR, Ghooshchi F, Moghaddam HRT, Kasraie P, and Oveysi M. 2023. Mitigation the adverse effects of salinity on red bean plants via exogenous application of glycine betaine, zinc, and manganese: physiological and morphological approach. *Russian Journal of Plant Physiology* 70(3):51. DOI: 10.1134/s1021443723600393

Estaji A, Kalaji HM, Karimi HR, Roosta HR, and Moosavi-Nezhad SM. 2019. How glycine betaine induces tolerance of cucumber plants to salinity stress? *Photosynthetica* 57(3):753-761. DOI: 10.32615/ps.2019.053

García-Caparrós P, Llanderal A, Hegarat E, Jiménez-Lao M, and Lao MT. 2020. Effects of exogenous application of osmotic adjustment substances on growth, pigment concentration, and physiological parameters of *Dracaena sanderiana* sander under different levels of salinity. *Agronomy* 10(1):125. DOI: 10.3390/agronomy10010125

Ghorbani A, Ghasemi-Omran VO, and Chen M. 2023. The effect of glycine betaine on nitrogen and polyamine metabolisms, expression of glycoside-related biosynthetic enzymes, and K/Na balance of stevia under salt stress. *Plants-Basel* 12(8):1628. DOI: 10.3390/plants12081628

Gibon Y, Bessieres MA, and Larher F. 1997. Is glycine betaine a non‐compatible solute in higher plants that do not accumulate it? *Plant Cell & Environment* 20(3):329-340. DOI: 10.1046/j.1365-3040.1997.d01-82.x

Graziani G, Cirillo A, Giannini P, Conti S, El-Nakhel C, Rouphael Y, Ritieni A, and Di Vaio C. 2022. Biostimulants improve plant growth and bioactive compounds of young olive trees under abiotic stress conditions. *Agriculture* 12(2):227. DOI: 10.3390/agriculture12020227

Guan G, Wang Y, Cheng H, Jiang Z, and Fei J. 2015. Physiological and biochemical response to drought stress in the leaves of *Aegiceras corniculatum* and *Kandelia obovata*. *Ecotoxicology* 24(7-8):1668-1676. DOI: 10.1007/s10646-015-1470-4

Habib N, Ashraf M, Ali Q, and Perveen R. 2012. Response of salt stressed okra (*Abelmoschus esculentus* moench) plants to foliar-applied glycine betaine and glycine betaine containing sugarbeet extract. *South African Journal of Botany* 83151-158. DOI: 10.1016/j.sajb.2012.08.005

Habibi F, Guillén F, Serrano M, and Valero D. 2022. Postharvest treatment with glycine betaine enhances chilling tolerance of blood orange fruit by increasing antioxidant defence systems and osmoregulation during cold storage. *Scientia Horticulturae* 305111352. DOI: 10.1016/j.scienta.2022.111352

Hafez EM, Gowayed SM, Nehela Y, Sakran RM, Rady AMS, Awadalla A, Omara AE, and Alowaiesh BF. 2021. Incorporated biochar-based soil amendment and exogenous glycine betaine foliar application ameliorate rice (*Oryza sativa* L.) Tolerance and resilience to osmotic stress. *Plants-Basel* 10(9):1930. DOI: 10.3390/plants10091930

Haghighi P, Habibi D, Mozafari H, Sani B, and Sadeghishoae M. 2021. Impact of methanol and glycine betaine on yield and quality of fodder beet genotypes (*Beta vulgaris* subsp. Vulgaris). *Agronomy* 11(11):2122. DOI: 10.3390/agronomy11112122

Hamani AKM, Chen J, Soothar MK, Wang G, Shen X, Gao Y, and Qiu R. 2021. Application of exogenous protectants mitigates salt-induced Na^+^ toxicity and sustains cotton (*Gossypium hirsutum* L.) seedling growth: comparison of glycine betaine and salicylic acid. *Plants (Basel, Switzerland)* 10(2):380. DOI: 10.3390/plants10020380

Hamani AKM, Li S, Chen J, Amin AS, Wang G, Xiaojun S, Zain M, and Gao Y. 2021. Linking exogenous foliar application of glycine betaine and stomatal characteristics with salinity stress tolerance in cotton (*Gossypium hirsutum* L.) Seedlings. *Bmc Plant Biology* 21(1):146. DOI: 10.1186/s12870-021-02892-z

Hamani AKM, Wang G, Soothar MK, Shen X, Gao Y, Qiu R, and Mehmood F. 2020. Responses of leaf gas exchange attributes, photosynthetic pigments and antioxidant enzymes in NaCl-stressed cotton (*Gossypium hirsutum* L.) seedlings to exogenous glycine betaine and salicylic acid. *Bmc Plant Biology* 20(1):434. DOI: 10.1186/s12870-020-02624-9

Hameed A, Hussain T, Gulzar S, Aziz I, Gul B, and Khan MA. 2012. Salt tolerance of a cash crop halophyte *Suaeda fruticosa*: biochemical responses to salt and exogenous chemical treatments. *Acta Physiologiae Plantarum* 34(6):2331-2340. DOI: 10.1007/s11738-012-1035-6

Hasanuzzaman M, Nahar K, Khan MIR, Mahmud JA, Alam MM, and Fujita M. 2019. Regulation of reactive oxygen species metabolism and glyoxalase systems by exogenous osmolytes confers thermotolerance in *Brassica napus*. *Gesunde Pflanzen* 72(1):3-16. DOI: 10.1007/s10343-019-00476-4

Hassine AB, Ghanem ME, Bouzid S, and Lutts S. 2008. An inland and a coastal population of the mediterranean xero-halophyte species *Atriplex halimus* L. differ in their ability to accumulate proline and glycinebetaine in response to salinity and water stress. *Journal of Experimental Botany* 59(6):1315-1326. DOI: 10.1093/jxb/ern040

Hoque MA, Banu MNA, Okuma E, Amako K, Nakamura Y, Shimoishi Y, and Murata Y. 2007. Exogenous proline and glycinebetaine increase NaCl-induced ascorbate–glutathione cycle enzyme activities, and proline improves salt tolerance more than glycinebetaine in tobacco bright yellow-2 suspension-cultured cells. *Journal of Plant Physiology* 164(11):1457-1468. DOI: 10.1016/j.jplph.2006.10.004

Hoque MA, Okuma E, Banu MNA, Nakamura Y, Shimoishi Y, and Murata Y. 2007. Exogenous proline mitigates the detrimental effects of salt stress more than exogenous betaine by increasing antioxidant enzyme activities. *Journal of Plant Physiology* 164(5):553-561. DOI: 10.1016/j.jplph.2006.03.010

Hu LX, Hu T, Zhang XZ, Pang HC, and Fu JM. 2012. Exogenous glycine betaine ameliorates the adverse effect of salt stress on perennial ryegrass. *Journal of the American Society for Horticultural Science* 137(1):38-46. DOI: 10.21273/JASHS.137.1.38

Huang S, Zheng X, Yao L, Luo L, Zuo T, Hou Q, and Ni W. 2022. Glycinebetaine facilitates the photosynthesis of albino tea under low temperature by regulating related gene methylation. *Scientia Horticulturae* 303111235. DOI: 10.1016/j.scienta.2022.111235

Hussain M, Malik MA, Farooq M, Ashraf MY, and Cheema MA. 2008. Improving drought tolerance by exogenous application of glycinebetaine and salicylic acid in sunflower. *Journal of Agronomy and Crop Science* 194(3):193-199. DOI: 10.1111/j.1439-037x.2008.00305.x

Ibrahim M, Anjum A, Khaliq N, Iqbal M, and Athar H. 2006. Four foliar applications of glycinebetaine did not alleviate adverse effects of salt stress on growth of sunflower. *Pakistan Journal of Botany* 38(5):1561-1570. https://www.pakbs.org/pjbot/PDFs/38(5)/PJB38(5)1561.pdf

Iqbal N, Ashraf M, and Ashraf MY. 2008. Glycinebetaine, an osmolyte of interest to improve water stress tolerance in sunflower (*Helianthus annuus* L.): Water relations and yield. *South African Journal of Botany* 74(2):274-281. DOI: 10.1016/j.sajb.2007.11.016

Iqbal N, Ashraf M, and Ashraf MY. 2009. Influence of exogenous glycine betaine on gas exchange and biomass production in sunflower (*Helianthus annuus* L.) under water limited conditions. *Journal of Agronomy and Crop Science* 195(6):420-426. DOI: 10.1111/j.1439-037x.2009.00381.x

Iqbal N, Ashraf Y, and Ashraf M. 2010. Modulation of endogenous levels of some key organic metabolites by exogenous application of glycine betaine in drought stressed plants of sunflower (*Helianthus annuus* L.). *Plant Growth Regulation* 63(1):7-12. DOI: 10.1007/s10725-010-9506-5

Islam MM, Hoque MA, Okuma E, Banu MNA, Shimoishi Y, Nakamura Y, and Murata Y. 2009. Exogenous proline and glycinebetaine increase antioxidant enzyme activities and confer tolerance to cadmium stress in cultured tobacco cells. *Journal of Plant Physiology* 166(15):1587-1597. DOI: 10.1016/j.jplph.2009.04.002

Islam S, Parrey ZA, Shah SH, and Mohammad F. 2021. Glycine betaine mediated changes in growth, photosynthetic efficiency, antioxidant system, yield and quality of mustard. *Scientia Horticulturae* 285110170. DOI: 10.1016/j.scienta.2021.110170

Jabeen N, Abbas Z, Iqbal M, Rizwan M, Jabbar A, Farid M, Ali S, Ibrahim M, and Abbas F. 2015. Glycinebetaine mediates chromium tolerance in mung bean through lowering of Cr uptake and improved antioxidant system. *Archives of Agronomy and Soil Science* 62(5):648-662. DOI: 10.1080/03650340.2015.1082032

Jalal-Ud-Din, Khan SU, Khan A, and Naveed S. 2015. Effect of exogenously applied kinetin and glycinebetaine on metabolic and yield attributes of rice (*Oryza sativa* L.) under drought stress. *Emirates Journal of Food and Agriculture* 27(1):75-81. DOI: 10.9755/ejfa.v27il.17950

Jiménez-Arias D, Hernándiz AE, Morales-Sierra S, García-García AL, García-Machado FJ, Luis JC, and Borges AA. 2022. Applying biostimulants to combat water deficit in crop plants: research and debate. *Agronomy* 12(3):571. DOI: 10.3390/agronomy12030571

Kayak N, Kal Ü, Dal Y, Yavuz D, and Seymen M. 2022. Do proline and glycine betaine mitigate the adverse effects of water stress in spinach? *Gesunde Pflanzen* 75(1):97-113. DOI: 10.1007/s10343-022-00675-6

Khalifa GS, Abdelrassoul M, Hegazi AM, and Elsherif MH. 2016. Attenuation of negative effects of saline stress in two lettuce cultivars by salicylic acid and glycine betaine. *Gesunde Pflanzen* 68(4):177-189. DOI: 10.1007/s10343-016-0376-2

Kim MC, Kim NS, Kim YB, Kim CM, Chung YS, and Park SU. 2020. Betaine hydrochloride treatment affects growth and phenylpropanoid accumulation in tartary buckwheat (*Fagopyrum tataricum*) seedlings under salt stress. *Agronomy* 10(6):906. DOI: 10.3390/agronomy10060906

Korkmaz A, and _irikçi R. 2011. Improving salinity tolerance of germinating seeds by exogenous application of glycinebetaine in pepper. *Seed Science and Technology* 39(2):377-388. DOI: 10.15258/sst.2011.39.2.10

Küçükyumuk Z. 2021. Foliarly applied osmotic preservative contributes to pear (*Pyrus comminus*) leaf and root nutritional status under drought stress. *Applied Ecology and Environmental Research* 19(4):3019-3028. DOI: 10.15666/aeer/1904_30193028

Kumar P. 2021. Soil applied glycine betaine with arbuscular mycorrhizal fungi reduces chromium uptake and ameliorates chromium toxicity by suppressing the oxidative stress in three genetically different sorghum (*Sorghum bicolor* L.) Cultivars. *Bmc Plant Biology* 21(1):336. DOI: 10.1186/s12870-021-03113-3

Larher FR, Aziz A, Gibon Y, Trotel-Aziz P, Sulpice R, and Bouchereau A. 2003. An assessment of the physiological properties of the so-called compatible solutes using in vitro experiments with leaf discs. *Plant Physiology and Biochemistry* 41(6-7):657-666. DOI: 10.1016/S0981-9428(03)00076-7

Li M, Guo S, Xu Y, Meng Q, Li G, and Yang X. 2014. Glycine betaine‐mediated potentiation of HSP gene expression involves calcium signaling pathways in tobacco exposed to NaCl stress. *Physiologia Plantarum* 150(1):63-75. DOI: 10.1111/ppl.12067

Li M, Wang G, Li J, and Cao F. 2016. Foliar application of betaine alleviates cadmium toxicity in maize seedlings. *Acta Physiologiae Plantarum* 38(4):95. DOI: 10.1007/s11738-016-2116-8

Li S, Li F, Wang J, Zhang W, Meng Q, Chen THH, Murata N, and Yang X. 2011. Glycinebetaine enhances the tolerance of tomato plants to high temperature during germination of seeds and growth of seedlings. *Plant, Cell & Environment* 34(11):1931-1943. DOI: 10.1111/j.1365-3040.2011.02389.x

Li ZG, and Zhu LP. 2014. Hydrogen sulfide donor sodium hydrosulfide-induced accumulation of betaine is involved in the acquisition of heat tolerance in maize seedlings. *Brazilian Journal of Botany* 38(1):31-38. DOI: 10.1007/s40415-014-0106-x

Liu NQ, Lin SY, and Huang BR. 2017. Differential effects of glycine betaine and spermidine on osmotic adjustment and antioxidant defense contributing to improved drought tolerance in creeping bentgrass. *Journal of the American Society for Horticultural Science* 142(1):20-26. DOI: 10.21273/JASHS03962-16

Lou YH, Sun X, Chao Y, Han F, Sun MJ, Wang TT, Wang H, Song FP, and Zhuge YP. 2019. Glycinebetaine application alleviates salinity damage to antioxidant enzyme activity in alfalfa. *Pakistan Journal of Botany* 51(1):19-25. DOI: 10.30848/PJB2019-1(3)

Ma Q, Wang W, Li Y, Li D, and Zou Q. 2006. Alleviation of photoinhibition in drought-stressed wheat (*Triticum aestivum*) by foliar-applied glycinebetaine. *Journal of Plant Physiology* 163(2):165-175. DOI: 10.1016/j.jplph.2005.04.023

Mahouachi J, Argamasilla R, and Gómez-Cadenas A. 2011. Influence of exogenous glycine betaine and abscisic acid on papaya in responses to water-deficit stress. *Journal of Plant Growth Regulation* 31(1):1-10. DOI: 10.1007/s00344-011-9214-z

Makela P, Mantila J, Hinkkanen R, Pehu E, and Peltonensainio P. 1996. Effect of foliar applications of glycinebetaine on stress tolerance, growth, and yield of spring cereals and summer turnip rape in Finland. *Journal of Agronomy and Crop Science-Zeitschrift Fur Acker Und Pflanzenbau* 176(4):223-234. DOI: 10.1111/j.1439-037X.1996.tb00467.x

Mäkelä P, Munns R, Colmer TD, Condon AG, and Peltonen-Sainio P. 1998. Effect of foliar applications of glycinebetaine on stomatal conductance, abscisic acid and solute concentrations in leaves of salt- or drought-stressed tomato. *Functional Plant Biology* 25(6):655. DOI: 10.1071/pp98024

Makela P, Peltonensainio P, Jokinen K, Pehu E, Setala H, Hinkkanen R, and Somersalo S. 1996. Uptake and translocation of foliar-applied glycinebetaine in crop plants. *Plant Science* 121(2):221-230. DOI: 10.1016/S0168-9452(96)04527-X

Maqsood MF, Shahbaz M, Arfan M, and Basra SMA. 2020. Presowing seed treatment with glycine betaine confers NaCl tolerance in quinoa by modulating some physiological processes and antioxidant machinery. *Turkish Journal of Botany* 45(1):1-14. DOI: 10.3906/bot-2009-13

Metwaly EE, Al-Yasi HM, Ali EF, Farouk HA, and Farouk S. 2022. Deteriorating harmful effects of drought in cucumber by spraying glycinebetaine. *Agriculture* 12(12):2166. DOI: 10.3390/agriculture12122166

Mickelbart MV, Chapman P, and Collier-Christian L. 2006. Endogenous levels and exogenous application of glycinebetaine to grapevines. *Scientia Horticulturae* 111(1):7-16. DOI: 10.1016/j.scienta.2006.07.031

Mickelbart MV, and Boine B. 2020. Glycinebetaine enhances osmotic adjustment of ryegrass under cold temperatures. *Agronomy* 10(2):210. DOI: 10.3390/agronomy10020210

Min K, Cho Y, Kim E, Lee M, and Lee S. 2021. Exogenous glycine betaine application improves freezing tolerance of cabbage (*Brassica oleracea* L.) Leaves. *Plants-Basel* 10(12):2821. DOI: 10.3390/plants10122821

Mohammed AR, and Tarpley L. 2009. High nighttime temperatures affect rice productivity through altered pollen germination and spikelet fertility. *Agricultural and Forest Meteorology* 149(6-7):999-1008. DOI: 10.1016/j.agrformet.2008.12.003

Mohammed AR, and Tarpley L. 2009. Impact of high nighttime temperature on respiration, membrane stability, antioxidant capacity, and yield of rice plants. *Crop Science* 49(1):313-322. DOI: 10.2135/cropsci2008.03.0161

Mohsen J, Saeed Y, Shahryar D, and Naser S. 2017. Effects of exogenous application of nano particles and compatible organic solutes on sunflower (*Helianthus annuus* L.). *Botanica Serbica* 41(1):37-46. DOI: 10.5281/zenodo.453554

Molla MR, Ali MR, Hasanuzzaman M, Al-Mamun MH, Ahmed A, Nazim-Ud-Dowla M, and Rohman MM. 2014. Exogenous proline and betaine-induced upregulation of glutathione transferase and glyoxalase I in lentil (*Lens culinaris*) under drought stress. *Notulae Botanicae Horti Agrobotanici Cluj-Napoca* 42(1):73-80. DOI: 10.15835/nbha4219324

Nawaz K, and Ashraf M. 2007. Improvement in salt tolerance of maize by exogenous application of glycinebetaine: growth and water relations. *Pakistan Journal of Botany* 39(5):1647-1653. https://pakbs.org/pjbot/PDFs/39(5)/PJB39(5)1647.pdf

Nazar Z, Akram NA, Saleem MH, Ashraf M, Ahmed S, Ali S, Alsahli AA, and Alyemeni MN. 2020. Glycinebetaine-induced alteration in gaseous exchange capacity and osmoprotective phenomena in safflower (C*arthamus tinctorius* L.) under water deficit conditions. *Sustainability* 12(24):10649. DOI: 10.3390/su122410649

Noreen S, Zafar ZU, Hussain K, Athar H, and Ashraf M. 2015. Assessment of economic benefits of foliarly applied osmoprotectants in alleviating the adverse effects of water stress on growth and yield of cotton (*Gossypium hirsutum* L.). *Pakistan Journal of Botany* 47(6):2223-2230. https://www.pakbs.org/pjbot/PDFs/47(6)/21.pdf

Nusrat N, Shahbaz M, and Perveen S. 2014. Modulation in growth, photosynthetic efficiency, activity of antioxidants and mineral ions by foliar application of glycinebetaine on pea (*Pisum sativum* L.) Under salt stress. *Acta Physiologiae Plantarum* 36(11):2985-2998. DOI: 10.1007/s11738-014-1670-1

Okuma E, Murakami Y, Shimoishi Y, Tada M, and Murata Y. 2004. Effects of exogenous application of proline and betaine on the growth of tobacco cultured cells under saline conditions. *Soil Science and Plant Nutrition* 50(8):1301-1305. DOI: 10.1080/00380768.2004.10408608

Oukarroum A, El Madidi S, and Strasser RJ. 2012. Exogenous glycine betaine and proline play a protective role in heat-stressed barley leaves (*Hordeum vulgare* L.): A chlorophyll*a* fluorescence study. *Plant Biosystems - An International Journal Dealing with All Aspects of Plant Biology* 146(4):1037-1043. DOI: 10.1080/11263504.2012.697493

Park E, Jeknic Z, and Chen THH. 2006. Exogenous application of glycinebetaine increases chilling tolerance in tomato plants. *Plant and Cell Physiology* 47(6):706-714. DOI: 10.1093/pcp/pcj041

Park EJ, Jeknic Z, Sakamoto A, Denoma J, Yuwansiri R, Murata N, and Chen T. 2004. Genetic engineering of glycinebetaine synthesis in tomato protects seeds, plants, and flowers from chilling damage. *Plant Journal* 40(4):474-487. DOI: 10.1111/j.1365-313X.2004.02237.x

Patade VY, Lokhande VH, and Suprasanna P. 2013. Exogenous application of proline alleviates salt induced oxidative stress more efficiently than glycine betaine in sugarcane cultured cells. *Sugar Tech* 16(1):22-29. DOI: 10.1007/s12355-013-0261-6

Quan J, Zheng W, Wu M, Shen Z, Tan J, Li Z, Zhu B, Hong S, Zhao Y, Zhu Z, and Zang Y. 2022. Glycine betaine and β-aminobutyric acid mitigate the detrimental effects of heat stress on Chinese cabbage (*Brassica rapa* L. ssp. *pekinensis*) seedlings with improved photosynthetic performance and antioxidant system. *Plants-Basel* 11(9):1213. DOI: 10.3390/plants11091213

Rady MOA, Semida WM, El-Mageed TAA, Hemida KA, and Rady MM. 2018. Up-regulation of antioxidative defense systems by glycine betaine foliar application in onion plants confer tolerance to salinity stress. *Scientia Horticulturae* 240614-622. DOI: 10.1016/j.scienta.2018.06.069

Rahman S, Miyake H, and Takeoka Y. 2015. Effects of exogenous glycinebetaine on growth and ultrastructure of salt-stressed rice seedlings (*Oryza sativa* L.). *Plant Production Science* 5(1):33-44. DOI: 10.1626/pps.5.33

Rasheed R, Ashraf MA, Hussain I, Haider MZ, Kanwal U, and Iqbal M. 2014. Exogenous proline and glycinebetaine mitigate cadmium stress in two genetically different spring wheat (*Triticum aestivum* L.) Cultivars. *Brazilian Journal of Botany* 37(4):399-406. DOI: 10.1007/s40415-014-0089-7

Raza MAS, Saleem MF, Shah GM, Khan IH, and Raza A. 2014. Exogenous application of glycinebetaine and potassium for improving water relations and grain yield of wheat under drought. *Journal of Soil Science and Plant Nutrition* 14(ahead):0-0. DOI: 10.4067/s0718-95162014005000028

Reddy KR, Seepaul R, Henry WB, Gajanayake B, Lokhande S, and Brand D. 2014. Maize (*Zea mays* L.) Yield and aflatoxin accumulation responses to exogenous glycinebetaine application. *International Journal of Plant Production* 8(2):271-289. DOI: 10.22069/IJPP.2014.1529

Rezaei MA, Jokar I, Ghorbanli M, Kaviani B, and Kharabian-Masouleh A. 2012. Morpho-physiological improving effects of exogenous glycine betaine on tomato (*Lycopersicum esculentum* Mill.) Cv. Ps under drought stress conditions. *Plant Omics* 5(2):79-86. https://www.pomics.com/kaviani_5_2_2012_79_86.pdf

Rezaei MA, Kaviani B, and Masouleh AK. 2012. The effect of exogenous glycine betaine on yield of soybean [*Glycine max* (L.) Merr.] In two contrasting cultivars pershing and dpx under soil salinity stress. *Plant Omics* 5(2):87-93. https://www.pomics.com/kaviani215_5_2_2012_87_93.pdf

Roussos PA, Ntanos E, Tsafouros A, and Denaxa NK. 2020. Strawberry physiological and biochemical responses to chilling and freezing stress and application of alleviating factors as countermeasures. *Journal of Berry Research* 10(3):437-457. DOI: 10.3233/jbr-190494

Saeed HM, Mirza JI, and Anjum MA. 2016. Glycinebetaine-induced modulation in some biochemical and physiological attributes of okra under salt stress. *Pakistan Journal of Botany* 48(6):2205-2210. DOI: 10.3906/bot-1304-65

Safdari-Monfared N, Noor-Mohammadi G, Shirani-Rad A, and Majidi-Heravan E. 2020. Effects of sowing date and glycine betaine application on yield components and oil yield in canola (*Brassica napus* L.). *Turkish Journal of Field Crops* 25(1):32-40. DOI: 10.17557/tjfc.737486

Sakr MT, El-Sarkassy NM, and Fuller MP. 2012. Osmoregulators proline and glycine betaine counteract salinity stress in canola. *Agronomy for Sustainable Development* 32(3):747-754. DOI: 10.1007/s13593-011-0076-3

Serapicos M, Afonso S, Gonçalves B, and Silva AP. 2022. Exogenous application of glycine betaine on sweet cherry tree (*Prunus avium* L.): Effects on tree physiology and leaf properties. *Plants-Basel* 11(24):3470. DOI: 10.3390/plants11243470

Seymen M, ahin A, and Tanrıverdi ÖB. 2023. Mitigation effects of proline and glycine betaine to green onion under flooding stress. *Gesunde Pflanzen* 75(5):1639-1652. DOI: 10.1007/s10343-023-00854-z

Shabala L, Mackay A, Tian Y, Jacobsen SE, Zhou D, and Shabala S. 2012. Oxidative stress protection and stomatal patterning as components of salinity tolerance mechanism in quinoa (*Chenopodium quinoa*). *Physiologia Plantarum* 146(1):26-38. DOI: 10.1111/j.1399-3054.2012.01599.x

Shafiq S, Akram NA, Ashraf M, García-Caparrós P, Ali OM, and Latef AAHA. 2021. Influence of glycine betaine (natural and synthetic) on growth, metabolism and yield production of drought-stressed maize (*Zea mays* L.) Plants. *Plants-Basel* 10(11):2540. DOI: 10.3390/plants10112540

Shah NA, Ullah S, Nafees M, and Khan MN. 2023. Exogenous effect of sugar beet extract on physio-biochemical traits of *Hordeum vulgare* L. Under induced salinity stress. *Gesunde Pflanzen* 75(6):2655-2667. DOI: 10.1007/s10343-023-00894-5

Shahbaz M, Masood Y, Perveen S, and Ashraf M. 2011. Is foliar-applied glycinebetaine effective in mitigating the adverse effects of drought stress on wheat (*Triticum aestivum* L.)? *Journal of Applied Botany and Food Quality* 84(2):192-199. DOI:10.5073/JABFQ.2011.084.028

Shahzad K, Ali A, Ghani A, Khalid T, Jamil M, Bashir H, Hussain R, and Naveed NH. 2021. Exogenous proline and glycine betaine alleviate nickel toxicity in mung bean by modulating biochemical parameters and antioxidant system. *Fresenius Environmental Bulletin* 30(7A):9334-9340. https://www.pakbs.org/pjbot/papers/1677142322.pdf

Shahzad K, Ali A, Ghani A, Nadeem M, Khalid T, Nawaz S, Jamil M, and Anwar T. 2023. Exogenous application of proline and glycine betaine mitigates nickel toxicity in mung bean plants by up-regulating growth, physiological and yield attributes. *Pakistan Journal of Botany* 5527-32. DOI: 10.30848/PJB2023-SI(3)

Shams M, Yildirim E, Ekinci M, Turan M, Dursun A, Parlakova F, and Kul R. 2016. Exogenously applied glycine betaine regulates some chemical characteristics and antioxidative defence systems in lettuce under salt stress. *Horticulture, Environment, and Biotechnology* 57(3):225-231. DOI: 10.1007/s13580-016-0021-0

Sharma J, Kumar S, Kumar V, Singh P, Khyalia P, Verma S, Saini S, and Sharma A. 2023. Foliar application of glycine betaine to ameliorate lead toxicity in barley plants by modulating antioxidant enzyme activity and biochemical parameters. *Environmental Research Communications* 5(7):75002. DOI: 10.1088/2515-7620/acde38

Shemi R, Wang R, Gheith EMS, Hussain HA, Cholidah L, Zhang K, Zhang S, and Wang L. 2021. Role of exogenous-applied salicylic acid, zinc and glycine betaine to improve drought-tolerance in wheat during reproductive growth stages. *Bmc Plant Biology* 21(1):574. DOI: 10.1186/s12870-021-03367-x

Sofy MR, Elhawat N, and Alshaal T. 2020. Glycine betaine counters salinity stress by maintaining high k+/na+ ratio and antioxidant defense via limiting Na^+^ uptake in common bean (*Phaseolus vulgaris* L.). *Ecotoxicology and Environmental Safety* 200110732. DOI: 10.1016/j.ecoenv.2020.110732

Sorwong A, and Sakhonwasee S. 2015. Foliar application of glycine betaine mitigates the effect of heat stress in three marigold (*Tagetes erecta*) cultivars. *The Hortic J* 84(2):161-171. DOI: 10.2503/hortj.MI-038

Sulpice R, Gibon Y, Cornic G, and Larher FR. 2002. Interaction between exogenous glycine betaine and the photorespiratory pathway in canola leaf discs. *Physiologia Plantarum* 116(4):460-467. DOI: 10.1034/j.1399-3054.2002.1160404.x

Taskin S, and Ertan E. 2023. Exogenous applications of kaolin and glycine betaine increased the yield and quality of olive fruit and olive oil. *Erwerbs-Obstbau* 65(2):337-346. DOI: 10.1007/s10341-022-00822-3

Tisarum R, Theerawitaya C, Samphumphuang T, Singh HP, and Cha-Um S. 2019. Foliar application of glycinebetaine regulates soluble sugars and modulates physiological adaptations in sweet potato (*Ipomoea batatas*) under water deficit. *Protoplasma* 257(1):197-211. DOI: 10.1007/s00709-019-01429-4

Tisarum R, Theerawitaya C, Samphumphung T, Takabe T, and Cha-Um S. 2019. Exogenous foliar application of glycine betaine to alleviate water deficit tolerance in two Indica rice genotypes under greenhouse conditions. *Agronomy* 9(3):138. DOI: 10.3390/agronomy9030138

Torstensson A, Young JN, Carlson LT, Ingalls AE, and Deming JW. 2019. Use of exogenous glycine betaine and its precursor choline as osmoprotectants in antarctic sea‐ice diatoms^1^. *Journal of Phycology* 55(3):663-675. DOI: 10.1111/jpy.12839

Wahid A, and Shabbir A. 2005. Induction of heat stress tolerance in barley seedlings by pre-sowing seed treatment with glycinebetaine. *Plant Growth Regulation* 46(2):133-141. DOI: 10.1007/s10725-005-8379-5

Wang C, Ma XL, Hui Z, and Wang W. 2008. Glycine betaine improves thylakoid membrane function of tobacco leaves under low-temperature stress. *Photosynthetica* 46(3):400-409. DOI: 10.1007/s11099-008-0072-2

Wang N, Cao F, Richmond MEA, Qiu C, and Wu F. 2019. Foliar application of betaine improves water-deficit stress tolerance in barley (*Hordeum vulgare* L.). *Plant Growth Regulation* 89(1):109-118. DOI: 10.1007/s10725-019-00510-5

Wei D, Zhang T, Wang B, Zhang H, Ma M, Li S, Chen THH, Brestic M, Liu Y, and Yang X. 2022. Glycinebetaine mitigates tomato chilling stress by maintaining high-cyclic electron flow rate of photosystem i and stability of photosystem ii. *Plant Cell Reports* 41(4):1087-1101. DOI: 10.1007/s00299-022-02839-0

Wei D, Zhang W, Wang C, Meng Q, Li G, Chen THH, and Yang X. 2017. Genetic engineering of the biosynthesis of glycinebetaine leads to alleviate salt-induced potassium efflux and enhances salt tolerance in tomato plants. *Plant Science* 25774-83. DOI: 10.1016/j.plantsci.2017.01.012

Xin ZL, Gao M, Li SQ, Li SX, and Liang ZS. 2011. Modulation of plant growth, water status and antioxidantive system of two maize (*Zea may* L.) Cultivars induced by exogenous glycinebetaine under long term mild drought stress. *Pakistan Journal of Botany* 43(3):1587. https://www.pakbs.org/pjbot/PDFs/43(3)/PJB43(3)1587.pdf

Xing WB, and Rajashekar CB. 1999. Alleviation of water stress in beans by exogenous glycine betaine. *Plant Science* 148(2):185-192. DOI: 10.1016/S0168-9452(99)00137-5

Xing WB, and Rajashekar CB. 2001. Glycine betaine involvement in freezing tolerance and water stress in *Arabidopsis thaliana*. *Environmental and Experimental Botany* 46(1):21-28. DOI: 10.1016/S0098-8472(01)00078-8

Yang X, and Lu C. 2006. Effects of exogenous glycinebetaine on growth, CO_2_ assimilation, and photosystem ii photochemistry of maize plants. *Physiologia Plantarum* 127(4):593-602. DOI: 10.1111/j.1399-3054.2006.00687.x

Yang Y, Huang C, Ge Z, Zhou B, Su G, Liu C, and Fei Y. 2022. Exogenous glycine betaine reduces drought damage by mediating osmotic adjustment and enhancing antioxidant defense in *Phoebe hunanensis*. *Phyton-Annales Rei Botanicae* 91(1):129-148. DOI: 10.32604/phyton.2022.016704

Yang ZM, Yu JJ, Merewitz E, and Huang BR. 2012. Differential effects of abscisic acid and glycine betaine on physiological responses to drought and salinity stress for two perennial grass species. *Journal of the American Society for Horticultural Science* 137(2):96-106. DOI: 10.21273/JASHS.137.2.96

Yaqoob H, Akram NA, Iftikhar S, Ashraf M, Khalid N, Sadiq M, Alyemeni MN, Wijaya L, and Ahmad P. 2019. Seed pretreatment and foliar application of proline regulate morphological, physio-biochemical processes and activity of antioxidant enzymes in plants of two cultivars of quinoa (*Chenopodium quinoa* Willd.). *Plants-Basel* 8(12):588. DOI: 10.3390/plants8120588

Yildirim E, Ekinci M, Turan M, Dursun A, Kul R, and Parlakova F. 2015. Roles of glycine betaine in mitigating deleterious effect of salt stress on lettuce (*Lactuca sativa* L.). *Archives of Agronomy and Soil Science* 61(12):1673-1689. DOI: 10.1080/03650340.2015.1030611

Youssef SM, Elhady SAA, Aref RM, and Riad GS. 2018. Salicylic acid attenuates the adverse effects of salinity on growth and yield and enhances peroxidase isozymes expression more competently than proline and glycine betaine in cucumber plants. *Gesunde Pflanzen* 70(2):75-90. DOI: 10.1007/s10343-017-0413-9

Zhang G, Ba Q, Chen S, Liu F, and Li G. 2020. Exogenous application of glycine betaine alleviates cadmium toxicity in super black waxy maize by improving photosynthesis, the antioxidant system and glutathione-ascorbic acid cycle metabolites. *Cereal Research Communications* 48(4):449-458. DOI: 10.1007/s42976-020-00062-9

Zhang L, Lai J, Gao M, and Ashraf M. 2013. Exogenous glycinebetaine and humic acid improve growth, nitrogen status, photosynthesis, and antioxidant defense system and confer tolerance to nitrogen stress in maize seedlings. *Journal of Plant Interactions* 9(1):159-166. DOI: 10.1080/17429145.2013.791379

Zhang LJ, Cisse EHM, Pu YJ, Miao LF, Xiang LS, Xu W, and Yang F. 2021. Potassium silicate combined with glycine betaine improved salt tolerance in *Dalbergia odorifera*. *Biologia Plantarum* 65323-332. DOI: 10.32615/bp.2021.044

Zhang LX, Lai JH, Liang ZS, and Ashraf M. 2014. Interactive effects of sudden and gradual drought stress and foliar‐applied glycinebetaine on growth, water relations, osmolyte accumulation and antioxidant defence system in two maize cultivars differing in drought tolerance. *J Agronomy Crop Science* 200(6):425-433. DOI: 10.1111/jac.12081

Zhang Q, Rue K, and Mueller J. 2014. The effect of glycinebetaine priming on seed germination of six turfgrass species under drought, salinity, or temperature stress. *Hortscience* 49(11):1454-1460. DOI: 10.21273/HORTSCI.49.11.1454

Zhang T, Zhang W, Li D, Zhou F, Chen X, Li C, Yu S, Brestic M, Liu Y, and Yang X. 2021. Glycinebetaine: a versatile protectant to improve rice performance against aluminium stress by regulating aluminium uptake and translocation. *Plant Cell Reports* 40(12):2397-2407. DOI: 10.1007/s00299-021-02780-8

Zhu M, Li Q, Zhang Y, Zhang M, and Li Z. 2022. Glycine betaine increases salt tolerance in maize (*Zea mays* L.) By regulating na+ homeostasis. *Frontiers in Plant Science* 13978304. DOI: 10.3389/fpls.2022.978304

Zouari M, Elloumi N, Labrousse P, Rouina BB, Abdallah FB, and Ahmed CB. 2018. Olive trees response to lead stress: exogenous proline provided better tolerance than glycine betaine. *South African Journal of Botany* 118158-165. DOI: 10.1016/j.sajb.2018.07.008

Zuzunaga-Rosas J, Silva-Valdiviezo D, Calone R, Lupuţ I, Ibáñez-Asensio S, Boscaiu M, Moreno-Ramón H, and Vicente O. 2023. Biochemical responses to salt stress and biostimulant action in tomato plants grown in two different soil types. *Horticulturae* 9(11):1209. DOI: 10.3390/horticulturae9111209

2. **CNKI literature:**

Ba QS, Zhang GS, Ling Y, Li GP, Song YX, Zhang PF, and Fu ZL. 2017. Physiological effects of root-applying glycine betaine on wheat seedlings growth under nickel stress. *Acta Botanica Boreali-Occidentalia Sinica* 37(2):315-320. DOI: 10.7606/j.issn.1000-4025.2017.02.0315

Ba QS, Zhang LL, Li GP, Liu Y, Zhang PF, Song YX, and Fu ZL. 2019. Effects of root-applying glycine betaine on reactive oxygen species metabolism of wheat roots under lead stress. *Journal of Yunnan University(Natural Sciences Edition)* 41(2):372-379. DOI: 10.7540/j.ynu.20170603

Ceng Q, Yang YC, Gong L, Jiang HW, and Zhang R. 2009. Influence of exogenous CaCl_2_ and glycinebetaine on seed germination of tobacco under NaCl stress. *Hunan Agricultural Sciences* 222(3):33-35. DOI: 10.3969/j.issn.1006-060X.2009.03.014

Ceng WJ, Chen FQ, Zhuang ZL, Ding YF, and Peng YL. 2020. Mitigation effect analysis of different exogenous regulatory substances on different drought-tolerance maize inbred lines under drought stress. *Agricultural Research in the Arid Areas* 38(3):55-63. DOI: 10.7606/j.issn.1000-7601.2020.03.08

Chen JF, Gu KY, Jia YH, Cheng X, Zou CM, and Xie XY. 2021. Effects of exogenous betaine and salicylic acid on the physiological characteristics of tobacco under manganese stress. *Acta Tabacaria Sinica* 27(2):79-86. DOI: 10.16472/j.chinatobacco.2020.243

Chen SX, Jia JJ, Wang WL, Ba QS, and Li GP. 2020. Effects of exogenous glycine betaine on rwactive oxygen species metabolism in super black waxy maize under cadmium stress. *Journal of Huaibei Normal University(Natural Science)* 41(1):54-58. DOI: 10.3969/j.issn.2095-0691.2020.01.011

Cui X, and Hua ZR. 2023. Effects of betaine on seed germination and seedling growth of *Verbena bonariensis* under salt stress. *Acta Agriculturae Jiangxi* 35(06):88-93. DOI: 10.19386/j.cnki.jxnyxb.2023.06.013

Dai HH, Shan YS, Xin ZQ, He X, and Wu NB. 2020. Effects of exogenous betaine on physiological characteristics and tropidine alkaloid content in *Atropa belladonna* L. Under NaCl stress. *Plant Science Journal* 38(3):400-409. DOI: 10.11913/PSJ.2095-0837.2020.30400

Dong J, Xing JC, Wang MW, Zhu XM, Ding HR, Liu C, Zhao BQ, Wen ZG, and Hong LZ. 2017. The effect of three exogenous substances on seed germination of *Portulaca oleracea* L. Under NaCl stress. *Jiangsu Agricultural Sciences* 45(14):103-106. DOI: 10.15889/j.issn.1002-1302.2017.14.029

Dong WK, Ma X, Zhou XW, Lu XP, Wang J, He J, Wang Y, and Ma HL. 2019. Effects of exogenous glycine betaine on the physiological characteristics in *Medicago sativa* seedlings under low-temperature stress. *Acta Agrestia Sinica* 27(1):130-140. DOI: 10.11733/j.issn.1007-0435.2019.01.017

Du D, Wang YN, Yu JZ, Gu JC, and Yang AZ. 2004. The effects of exogenous betaine on photosynthesis of peach leaves under water stress. *Journal of Beijing Agricultural College* 19(2):1-4, 34. DOI: 10.3969/j.issn.1002-3186.2004.02.001

Fan CL, and Luo Q. 2016. Effects of exogenous betaine on photosynthesis, osmotic regulation, and protective enzyme activity in *Punica granatum* L. under drought stress. *Jiangsu Agricultural Sciences* 44(11):229-232. DOI: 10.15889/j.issn.1002-1302.2016.11.068

Gao XP, Zhang J, Niu TH, Liu Y, Chang YL, Liu ST, and Jie JM. 2023. Effect of glycine betaine on physiological characteristics of eggplant seedlings under high temperature stress. *Acta Agriculturae Zhejiangensis* 35(9):2097-2108. DOI: 10.3969/j.issn.1004-1524.20221258

Gao Y, Li C, and Lou K. 2011. Effect of glycine betaine on physiological characteristics of cotton seedlings under drought stress. *Plant Nutrition and Fertilizer Science* 17(2):513-516. DOI: 10.11674/zwyf.2011.0355

Gao Y, Lou K, and Li C. 2011. Effect of exogenous glycine betaine on the physiological responses of cotton seedlings under salt stress. *Transactions of the Csae* 27(S1):244-248. http://www.tcsae.org/cn/article/pdf/preview/20111348.pdf

Gu WY, Mou YY, Qian Z, Xue Y, and Gao HW. 2013. The alleviating effect of exogenous betaine on mitochondrial membrane oxidative damage in *Cichorium intybus* L. Seedlings under salt stress. *Jiangsu Agricultural Sciences* 41(7):198-201. DOI: 10.3969/j.issn.1002-1302.2013.07.070

Gu WY, Qi XM, Li XZ, and Gao HW. 2012. Effects of exogenous glycine betaine on seeds germination & bud seedling growth of *Cichorium intybus* L. under salt stress. *Chinese Agricultural Science Bulletin* 28(29):10-14. DOI: 10.3969/j.issn.1000-6850.2012.29.003

Gu KY, Hou S, Chen JF, Liu RR, Zou CM, Xu ZL, and Xie XY. 2021. Effects of exogenous glycine betaine on the physiological characteristics of tobacco seedlings under low temperature stress. *Journal of Yunnan Agricultural University* 36(2):283-290. DOI: 10.12101/j.issn.1004-390X(n).201908036

Guo QF, Ma QQ, Sun C, Lu SZ, Zhang QM, and Wang W. 2004. Effects of exogenous glycinebetaine on the salt-tolerance of wheat. *Acta Botanica Boreali-Occidentalia Sinica* 24(9):1680-1686. DOI: 10.3321/j.issn:1000-4025.2004.09.021

Guo ZJ, Wang Y, He XM, Zhang XN, and Lu GH. 2017. Effect of root-applied glycinebetaine on glycinebetaine accumulation and salinity tolerance of seedling of *Populus euphratica*. *Arid Zone Research* 34(4):847-855. DOI: 10.13866/j.azr.2017.04.18

Han DH, Wang EJ, Zhang Y, Wang HX, Wang Y, and Wang F. 2021. Effects of exogenous spermidine and glycine betaine on seed germination and physiological characteristics of *Isatis indigotica* Fort.seedlings under drought stress. *Crops* 200(1):118-123. DOI: 10.16035/j.issn.1001-7283.2021.01.017

Han DH, Wang EJ, and Zhang Y. 2020. Effect of glycine betaine on seed germination and antioxidant enzymes in seedlings of *Astragalus membranaceus* under salt stress. *Fujian Journal of Agricultural Sciences* 35(8):891-895. DOI: 10.19303/j.issn.1008-0384.2020.08.012

Han ZP, Zhang HX, Chen ZY, and Zhang X. 2018. Effect of betaine on seed germination characteristics of cucumber under Ca(NO_3_)_2_ stress. *Seed* 37(2):24-28. DOI: 10.16590/j.cnki.1001-4705.2018.02.024

He LD, Liu GM, Yang JS, Lu ZZ, and Li JB. 2013. Effects of exogenous substances priming on *Suaeda salsa* seed germination under NaCl stress. *Pratacultural Science* 30(06):860-867. https://cykx.lzu.edu.cn/cn/article/pdf/preview/f92ad32f-6ff7-4f73-af9f-afecee7c9f98.pdf

Hou PF, Ma JQ, Zhao PF, Zhang HL, Zhao HJ, Liu HS, Zhao YD, and Wang YX. 2013. Effects of betaine on chloroplast protective enzymes and *psbA* gene expression in wheat seedlings under drought stress. *Acta Agronomica Sinica* 39(7):1319-1324. DOI: 10.3724/SP.J.1006.2013.01319

Hu Y, Liu JH, Wang P, and Ma HG. 2009. Physiological effects of glycine betaine under high temperature stress of *Phaseolus vulgaris* L. seedlings. *Northern Horticulture* 33(8):5-8. DOI: 10.11937/bfyy.200908002

Hua ZR. 2022. Effects of exogenous glycine betaine on *Lythrum salicaria* seed germination and seedling growth under salt stress. *Journal of Northwest A & F University(Nat. Sci. Ed.)* 50(09):119-126. DOI: 10.13207/j.cnki.jnwafu.2022.09.014

Hua ZR, Cui X, and Li XL. 2022. Effects of exogenous betaine on physiological characteristics of *Gentiana scabra* seedlings under salt stress. *Acta Agriculturae Jiangxi* 34(05):81-87. DOI: 10.19386/j.cnki.jxnyxb.2022.05.015

Hua ZR, Liu F, and Li XL. 2022. Effect of exogenous betaine on physiological characteristics of *Platycodon grandiflorum* seedlings under salt stress. *Acta Agriculturae Jiangxi* 34(06):67-72. DOI: 10.19386/j.cnki.jxnyxb.2022.06.012

Huang LH. 2006. Effect of exogenous betaine on cold resistance of corn seedlings. *Hubei Agricultural Sciences* 45(2):168-170. DOI: 10.3969/j.issn.0439-8114.2006.02.014

Huang YY, Zhu DH, Li HZ, and Dong DK. 2012. Effects of exogenous glycine betaine on the physiological indexes of soybean seedlings under NaCl stress. *Acta Agriculturae Zhejiangensis* 24(1):12-16. DOI: 10.3969/j.issn.1004-1524.2012.01.003

Hui HX, Xu X, and Li QR. 2003. Exogenous betaine improves photosynthesis of *Lycium barbarum* under salt stress. *Acta Botanica Boreali-Occidentalia Sinica* 23(12):2137-2142. DOI: 10.3321/j.issn:1000-4025.2003.12.015

Hui HX, Xu X, and Li QR. 2004. Effect of exogenous betaine on the growth and membrane lipid peroxidation in *Lycium barbarum* under salt stress. *Journal of Northwest A & F University(Natural Science Edition)* 32(07):77-80. DOI: 10.13207/j.cnki.jnwafu.2004.07.018

Jia ZG, Zhang L, Xin LB, and Yuan HF. 2017. Effects of exogenous substances on membrane lipid peroxidation of *Ephedra sinica* seedlings under drought stress. *Northern Horticulture* 41(1):158-163. DOI: 10.11937/bfyy.201701034

Jiang HL, Lai RF, Li SY, Wang M, Tang XR, Duan MY, Pan SG, Tian H, Wang SL, and Mo ZW. 2018. Effects of exogenous regulators on yield formation and transportation characteristics of stem and sheath matter in fragrant rice. *Journal of Southern Agriculture* 49(8):1505-1511. DOI: 10.3969/j.issn.2095-1191.2018.08.06

Jiang XM, Qin Y, Guo GZ, Chen DM, and Gu LPRS. 2013. The effects of exogenous substances on chilling resistance of pepper seedlings. *Chinese Agricultural Science Bulletin* 29(25):87-92. DOI: 10.3969/j.issn.1000-6850.2013.25.017

Jiang XM, Qin Y, Nu SRTK, and Guo GZ. 2014. Physiological response of pepper seedlings to low temperature stress under exogenous substance treatment. *Xinjiang Agricultural Sciences* 51(10):1821-1829. DOI: 10.6048/j.issn.1001-4330.2014.10.011

Jiang XM, Qin Y, and Guo GZ. 2013. Research on effects of exogenous substances on germination of pepper seed under low temperature stress. *Journal of Xinjiang Agricultural University* 36(3):229-233. DOI: 10.3969/j.issn.1007-8614.2013.03.011

Jiang YT, Qu GN, Guo Y, Pan LX, and Li YM. 2021. Effects of exogenous SA and GB on seed germination of *Impatiens balsamena* under NaCl stress. *Northern Horticulture* 45(5):77-83. DOI: 10.11937/bfyy.20201121

Jing RL, Chang XP, and Hu RH. 1998. Effects of exogenous betaine on the drought resistance of winter wheat seedlings. *Agricultural Reseach in the Arid Areas* 16(2):4-8. https://kns.cnki.net/kcms2/article/abstract?v=QLLcG2LOr2pgXWqCmilNzf-07vhuGZVqUGXN8-OGyZXepTYUuCrA3Kjm2sjktyXSrJwGYEh9bBk0tGYxzi9aeNWWVfh3N4CmF5UJp18Lw0_Co9pLQp-EFlzwpXtLXQ2O-X1ZgM3fuJ6Xmcqn0W2gwvYAZTPFx_Dkz-KGX78juAk=&uniplatform=NZKPT&language=CHS

Li AM, Zhang L, Zhang C, Zhao PB, Gou W, Chen FC, Gao M, and Zhang LX. 2017. Effects of fulvic acid and betaine pretreatment on physiological characteristics and photosynthesis of *Malus hupehensis* under drought stress. *Acta Botanica Boreali-Occidentalia Sinica* 37(2):307-314. DOI: 10.7606/j.issn.1000-4025.2017.02.0307

Li B, and Li SX. 2005. Effect of seed treatment with betaine on the salt resistance of maize seedlings. *Journal of Tianshui Normal University* 25(5):64-65. DOI: 10.3969/j.issn.1671-1351.2005.05.022

Li MF, Wei JX, Fu LF, and Li SP. 2011a. Effects of exogenous betaine on the endogenous betaine synthesis in banana under low temperature stress. *Acta Botanica Boreali-Occidentalia Sinica* 31(7):1400-1404. https://kns.cnki.net/kcms2/article/abstract?v=QLLcG2LOr2pE32XvuzyrzsimAU6_DjeQORxHB7hAQLyfflog1VDX-U4g6I96bVvg4cNqMHdEyQ1csNdLuf4jCelXe1eRtFXi5qWYXVeMsuajo1QhBZZW-AM7tPhCC8aPK3_4QsTqHVVYV6OHcRJ7qjer6Uh8eQAp5ZmuLYiw04VnUnAlG8cdUg==&uniplatform=NZKPT&language=CHS

Li MF, Wei JX, Fu LF, and Li SP. 2011b. Effects of exogenous betaine on the endogenous betaine synthesis in leaf and root of banana under low temperature stress. *Chinese Agricultural Science Bulletin* 27(16):244-247. DOI: [10.11924/j.issn.1000-6850.2011-0060](https://doi.org/10.11924/j.issn.1000-6850.2011-0060)

Li SJ, Han DH, Wang EJ, and Wu Y. 2016. Effects of exogenous betaine on seed germination and antioxidase activities of *Lycium ruthenium* seedlings under NaCl stress. *Pratacultural Science* 33(4):674-680. DOI: 10.11829/j.issn.1001-0629.2015-0404

Li WX, Yao TM, Wang P, Chen GL, and Huo SX. 2010. Effects of exogenous betaine on the seedlings growth of pumpkin under NaCl stress. *Agricultural Science & Technology* 11(02):106-108. DOI: 10.16175/j.cnki.1009-4229.2010.02.038

Li XL, He GX, and Hua ZR. 2021. Effects of exogenous osmotic adjustment substances on drought resistance of *Scutellaria baicalensis* seedlings. *Acta Agriculturae Jiangxi* 33(11):20-27. DOI: 10.19386/j.cnki.jxnyxb.2021.11.004

Li XL, and Hua ZR. 2019. Effect of glycine betaine on physiological property of *Scutellaria baicalensis* seedlings under low temperature stress. *Guizhou Agricultural Sciences* 47(3):119-123. DOI: 10.3969/j.issn.1001-3601.2019.03.027

Li YM, Yang JG, Zhang L, Liao SQ, Sun N, and Sun YX. 2020. Effects of spraying exogenous substance and irrigation on yield, quality and water use of lettuce. *Water Saving Irrigation* 4(8):44-49. DOI: 10.3969/j.issn.1007-4929.2020.08.009

Li YM, Zhou YW, Zhang L, Liao SQ, and Sun YX. 2021. Coupling effects of stress-resistant substances and osmotic regulators on tomato yield and water use efficiency and its possible mechanism. *Journal of Agricultural Science and Technology* 23(1):43-50. DOI: 10.13304/j.nykjdb.2019.0333

Li Y, Dong R, Liu ZM, Wang W, Jiang XM, and Yu XH. 2015. The effect of betaine on cold tolerance related indicators of cucumber seedlings under low temperature stress. *Jiangsu Agricultural Sciences* 43(11):217-218, 219. DOI: 10.15889/j.issn.1002-1302.2015.11.063

Li YY, Liang GJ, Li YH, and Ye QS. 2004. Effects of exogenous betaine on cold resistance of cucumber seedlings. *Plant Physiology Communications* 40(6):673-676. DOI: 10.13592/j.cnki.ppj.2004.06.005

Li ZG, Xu SL, Cui MK, Liu X, and Gong M. 2012. Remission effect of exogenous betaine on biological membrane injury of *Jatropha curcas* under peg stress. *Seed* 31(6):40-44. DOI: 10.3969/j.issn.1001-4705.2012.06.010

Liang TB, Zhang JL, Tian L, Wang JW, Yin QS, Cai XJ, Liu Y, and Zhang SX. 2013. Effects of exogenous glycine betaine and proline on antioxidant metabolism of flue-cured tobacco under drought stress. *Tobacco Science & Technology* 46(2):68-71. DOI: 10.3969/j.issn.1002-0861.2013.02.016

Liang XH, An MY, Song Z, Xu GC, and Pu YXH. 2015. Effects of exogenous glycine betaine on the physiological characteristics of *Zoysia japonica* under low-temperature stress. *Acta Prataculturae Sinica* 24(9):181-188. DOI: 10.11686/cyxb2014431

Lin ZL, Hao T, Yu JJ, and Yang ZM. 2022. Effects of three exogenous substances on the physiological indexes of tall fescue under salt stress. *Pratacultural Science* 39(4):720-730. DOI: 10.11829/j.issn.1001-0629.2021-0537

Liu CC, Li X, Ren SW, and Chen HN. 2019. The effect of exogenous betaine on the growth of rice seedlings under low temperature stress. *Jiangsu Agricultural Sciences* 47(11):100-102. DOI: 10.15889/j.issn.1002-1302.2019.11.021

Liu FF, Dong ST, Chen SX, Ba QS, and Li GP. 2019. The effect of root application of betaine on physiological traits of maize seedlings under cadmium stress. *Journal of Chifeng University(Natural Science Edition)* 35(08):36-37. DOI: 10.13398/j.cnki.issn1673-260x.2019.08.011

Liu H, Jing HJ, Zhou X, and Wang R. 2017. Effects of betaine on physiological characteristics of maize seedlings under Pb stress. *Journal of Fuyang Normal University(Natural Science)* 34(01):46-50. DOI: 10.14096/j.cnki.cn34-1069/n/1004-4329(2017)01-046-05

Liu J, Liu HP, and Liu YL. 2004. Effect of exogenous glycine betaine on polyamines and ions content of barley seedlings under salt stress. *Acta Agronomica Sinica* 30(11):1119-1123. DOI: https://zwxb.chinacrops.org/CN/Y2004/V30/I11/1119

Liu JY, Shi GL, and Cui HM. 2009. Influence of exogenous application of glycinebetaine on physiology characteristic of salt-stressed processing tomato plants. *Northern Horticulture* 33(8):243-246. DOI: 10.11937/bfyy.200908089

Liu LP, Wang JC, Si EJ, Yao LR, Lu ZH, Qi TT, Ma XL, Wang HJ, Li BC, Zhao CM, Shang XW, and Meng YX. 2023. Effect of exogenous betaine and proline on seed germination and seedling growth of barley under salt stress. *Journal of Triticeae Crops* 43(6):766-774. DOI: 10.7606/j.issn.1009-1041.2023.06.12

Liu RD, Wang YN, Wang LX, and Li LG. 2004. Effect of exogenous betaine on physiological index of drought resistance for apricot. *Journal of Inner Mongolia Agricultural University(Natural Science Edition)* 25(2):69-72. DOI: 10.3969/j.issn.1009-3575.2004.02.016

Liu RD, Wang YN, Yu TQ, Lu P, and Gu JC. 2004. Effect of exogenous betaine on photosynthesis of water stressed leaves of apricot. *Journal of Beijing Agricultural College* 19(1):10-13. DOI: 10.3969/j.issn.1002-3186.2004.01.004

Liu SJ, Liu AQ, and He M. 2018. Effect of exogenous glycine betaine on eggplant seedling resistance under low temperature stress. *Northern Horticulture* 42(22):57-61. DOI: 10.11937/bfyy.20173648

Liu SL, Yang P, and Yin SX. 2015. Regulatory role of exogenous glycine betaine on the drought tolerance of creeping bentgrass. *Acta Prataculturae Sinica* 24(3):80-88. DOI: 10.11686/cyxb20150308

Liu YT, Xu RX, Wang HT, Shi YJ, Li CY, Ma FW, and Liang W. 2022. Exogenous glycine betaine improved the resistance of apple to *Glomerella* leaf spot. *Journal of Fruit Science* 39(7):1252-1261. DOI: 10.13925/j.cnki.gsxb.20210566

Lu J, Xing XJ, Zhu LQ, Wang Y, Yin H, and Yuan JJ. 2011. Effects of exogenous glycine betaine and CaCl_2_ on physiological responses of tobacco plants under stresses of heat and drought. *Plant Nutrition and Fertilizer Science* 17(06):1437-1443. DOI: 10.11674/zwyf.2011.1054

Lu XP, Dong WK, Zhang R, Zhou XW, Wang J, He J, and Ma HL. 2019. Effect of exogenous betaine on seed germination and physiological characteristics of alfalfa seedlings under cadmium stress. *Grassland and Turf* 39(6):1-10. DOI: 10.13817/j.cnki.cyycp.2019.06.001

Luo Y, Wang YJ, Xie SL, Zhao XX, Yang XH, and Wang W. 2005. Effects of isotonic water and salt stress on seed germination of tobacco and protective function of exogenous glycinebetaine. *Acta Agronomica Sinica* 31(08):1029-1034. DOI: https://zwxb.chinacrops.org/CN/Y2005/V31/I08/1029

Ma MZ. 2016. Effects of exogenous glycinebetaine on the functions of apple saplings leaf photosystemⅱ,osmotic adjustment ability and protective enzyme activity under drought stress. *South China Fruits* 45(05):113-116. DOI: 10.13938/j.issn.1007-1431.20160178

Ma TY, and Li YZ. 2019. Effects of exogenous betaine on alfalfa seed germination and seedling resistance under NaCl stress. *Pratacultural Science* 36(12):3100-3110. DOI: 10.11829/j.issn.1001-0629.2019-0361

Ma ZL, Song J, and Li JJ. 2012. Effects of exogenous glycine betaine(GB) on cold resistance of *Trachelospermum Jasminoides* Flame and *T. Jasminoides* (Lindl.) Lem. *Journal of Anhui Agricultural Sciences* 40(2):678-679. DOI: 10.3969/j.issn.0517-6611.2012.02.018

Ma ZL, Zhou HF, Ran CY, He QL, Huang ZC, and Wang LC. 2019. Effects of exogenous glycine betaine and salicylic acid on physiological characters of hot pepper(*Capsicum annuum* L.) Under drought stress during blossom and fruit period. *Ecology and Environmental Monitoring of Three Gorges* 4(02):57-63. DOI: 10.19478/j.cnki.2096-2347.2019.02.08

Ma ZL, Zhou HF, and Xie XY. 2019. Effect of betaine(GB) and salicylic acid(SA) on phytosynthetic characters of hot pepper under drought stress during blossom and fruit period. *Northern Horticulture* 43(11):18-23. DOI: 10.11937/bfyy.20181415

Meng F, Yu SL, Zheng QQ, Liu DD, Li PC, and He J. 2008. Effects of exogenous glycine betaine on the heat resistance of young grape plants. *Journal of Fruit Science* 25(4):581-584. DOI: 10.3969/j.issn.1009-9980.2008.04.026

Mi YW, Chen Y, Guo FX, and Wang LQ. 2012. Physiological response of *Lycium ruthenicum* seedling to exogenous betaine under salt stress. *Pratacultural Science* 29(9):1417-1421. https://cykx.lzu.edu.cn/cn/article/pdf/preview/6f7aa9a8-8b8d-401b-9f78-f58fc3fd4526.pdf

Nu EMGL, A LMJK, Cui YN, Pan JJ, Lou SW, and Zhao Q. 2018. Effects of different exogenous substances on the agronomic and economic characteristics of cotton under drought stress. *Xinjiang Agricultural Sciences* 55(11):1977-1987. DOI: 10.6048/j.issn.1001-4330.2018.11.003

Pan LX, Chen CX, Wang H, Zheng T, Feng LM, and Jiang YT. 2020. The effect of exogenous substances on the germination of *Dianthus chinensis* L. seeds under salt stress. *Xiandai Nongcun Keji* 591(11):77-79. DOI: 10.3969/j.issn.1674-5329.2020.11.066

Quan J, Zhang LS, Li XW, Zhang HT, Zhang LX, Li BZ, and Ma FW. 2012. Effect of exogenous substances on antioxidant protection enzyme activity of drought stress and rewater for *Malus hupehensis*. *Northern Horticulture* 36(18):5-10. http://bfyy.paperonce.org/oa/DArticle.aspx?type=view&id=201218002

Ren XX, Wang HJ, and Yang HX. 2023. The alleviating effects of exogenous calcium and betaine on silk gourd seedlings under drought stress. *Jiangsu Agricultural Sciences* 51(8):131-138. DOI: 10.15889/j.issn.1002-1302.2023.08.019

Shi YF, Min WF, Bai XR, She YMF, Tian HT, and Luo CK. 2023. Effects of exogenous regulatory substances on physiological characteristics and gene expression of rice seedlings under alkali stress. *Plant Nutrition and Fertilizer Science* 29(5):813-825. DOI: 10.11674/zwyf.2022582

Su LY, Hu Y, Liang J, Yang ZQ, Chen X, and Sun MS. 2021. Effects of exogenous substances on physiological characteristics of *Michelia macclurei* under salt stress. *Journal of Northeast Forestry University* 49(7):16-21. DOI: 10.3969/j.issn.1000-5382.2021.07.003

Sun WY, Wang H, and Huang JC. 2001. The effect of external betaine on membrane lipid peroxidation of wheat seedling under water stress. *Acta Botanica Boreali-Occidentalia Sinica* 21(3):487-491. DOI: 10.3321/j.issn:1000-4025.2001.03.016

Sun XG. 2023. The effect of exogenous growth regulators on cotton yield formation. *Xinjiang Agricultural Science and Technology* 1(02):24-25. https://kns.cnki.net/kcms2/article/abstract?v=QLLcG2LOr2qUbTB6It3sivQGJtCekSx2waD1ZTslGboI8isaKMSjpX6XvOxnqrANrBrjFNguCefOxQzxKGY3hRs5y70pEa5CSI79FyzHH6lSy7o8E4UxTiZc6AoR7dEh_43szHoogkaF4DKetN0RMDHViI9VFgp5j39OZXoAMCP_dvcjQNx18g==&uniplatform=NZKPT&language=CHS

Tang L, Zhu BW, Qiao F, and Li XG. 2023. Effects of exogenous betaine on physiology of Brazil banana under osmotic stress. *Journal of Southwest Forestry University* 43(3):26-34. DOI: 10.11929/j.swfu.202111002

Tian YT, Gao TT, Wang XY, Luo SYZ, and Miao YJ. 2022. Effects of exogenous glycine betaine on seed germination of *Elymus nutans* Griseb under NaCl stress. *Journal of Plateau Agriculture* 6(01):1-10. DOI: 10.19707/j.cnki.jpa.2022.01.001

Wang BB, Li QG, Yu JJ, and Yang ZM. 2022. Effects of three exogenous substances on the salt tolerance of creeping bentgrass. *Pratacultural Science* 39(3):474-484. DOI: 10.11829/j.issn.1001-0629.2021-0330

Wang GP, Wang JZ, Xue XM, Lu C, and Nie PX. 2014. Effect of exogenous glycinebetaine on physiological indicators of *Malus hupehensis* leaves under drought stress. *Chinese Agricultural Science Bulletin* 30(22):128-134. DOI: 10.11924/j.issn.1000-6850.2014-0165

Wang GX, Lu C, Zhao Q, Yang YZ, Zheng JP, Li CG, and Zhang YL. 2020. Effects of exogenous glycine betaine on physiological characteristics of *Camellia oleifera* under low temperature stress. *Journal of Northwest Forestry University* 35(5):78-84. DOI: 10.3969/j.issn.1001-7461.2020.05.12

Wang J, He P, Zhang CP, Wei PX, and Duan CX. 2014. Effect of exogenous nitric oxide donor snp, betaine and humic acidon physiological characters of *Celosia cristata* seedlings under drought stress. *Journal of Southwest University(Natural Science Edition)* 36(04):14-21. DOI: 10.13718/j.cnki.xdzk.2014.04.003

Wang LP, Xue XM, Lu C, Nie PX, and Wang JZ. 2017. The effect of several stress enhancing agents on the cold resistance of apple flowers and young fruits. *Jiangsu Agricultural Sciences* 45(9):109-112. DOI: 10.15889/j.issn.1002-1302.2017.09.029

Wang LJ, Liang GS, Cheng FL, Zhang XY, Tang RY, and Li XW. 2023. The effect of different exogenous substances on the antioxidant capacity of pepper seedlings under sub low temperature conditions. *Agricultural Engineering Technology* 43(24):58-59. DOI: 10.16815/j.cnki.11-5436/s.2023.24.018

Wang SH, Zhu FM, Sun ML, Xu ZJ, Jiang XF, and Qiao F. 2020. Exogenous betaine modulating the tolerance of osmotic stress in watermelon cells. *Chinese Journal of Tropical Crops* 41(9):1816-1821. DOI: 10.3969/j.issn.1000-2561.2020.09.014

Wang YM, Wang YN, Shi GL, Du D, Liu RD, Yang AZ, Ge SL, and Meng HL. 2007. Effects of exogenous betaine on physiological responses of peach tree under water stress. *Chinese Journal of Applied Ecology* 18(3):542-548. DOI: https://www.cjae.net/CN/Y2007/V18/I03/542

Wang YB, Li B, Wei L, Yang YJ, Li SX, and Wang JP. 2005. Effects of exogenous betaine on salt-stress tolerance for two varieties in the leaves of maize seedlings. *Journal of Lanzhou University(Natural Sciences)* 41(2):34-37. DOI: 10.3321/j.issn:0455-2059.2005.02.008

Wang YJ, Hao XL, Zhou JJ, Guan Z, and Zhang FG. 2022. Effects of exogenous glycine betaine on seed germination and seedling growth of alfalfa under drought stress. *Northern Horticulture* 46(15):51-57. DOI: 10.11937/bfyy.20220600

Wei JX, Li SP, and Li MF. 2007. Effects of betaine on cold resistance of banana seedlings. *Guangdong Agricultural Sciences* 34(7):41-43. DOI: 10.3969/j.issn.1004-874X.2007.07.014

Wei SW, Wang HW, Zhang Y, and Wang SM. 2012. Effect of betaine on physiological indicators of pear tree under drought stress. *Shandong Agricultural Sciences* 44(6):50-52. DOI: 10.3969/j.issn.1001-4942.2012.06.012

Wu WJ, and Cai XX. 2009. Seedling growth of *Pisum sativum* in glycinebetaine and ZnSO_4_ under seawater. *Crops* 132(5):31-34. DOI: 10.3969/j.issn.1001-7283.2009.05.008

Wu WJ, and Wang JR. 2010. Alleviating effect of glycinebetaine and ZnSO_4_ on the seedling growth of *Lactuca sativa* under seawater stress. *Guangdong Agricultural Sciences* 37(2):42-45. DOI: 10.3969/j.issn.1004-874X.2010.02.015

Wu WJ, and Wang Q. 2010. Effect of exogenous matters on development of *Calendula officinalis* under seawater stress. *Guizhou Agricultural Sciences* 38(7):58-62. DOI: 10.3969/j.issn.1001-3601.2010.07.018

Xu T, Fan JS, Zhao BR, Lian YL, Qu ZC, and Li QC. 2016. Effect of glycine betaine on growth,yield and quality of melon under salt stress. *Heilongjiang Agricultural Sciences* 38(2):84-86. DOI: 10.11942/j.issn1002-2767.2016.02.0084

Xu T, Wang JQ, Han YH, Zhou C, Qu ZC, Han DW, Gao P, and Xu YY. 2023. Effects of exogenous glycine betaine seed soaking on maize seed germination under low temperature stress and seedling growth. *Heilongjiang Agricultural Sciences* 45(8):8-12. DOI: 10.11942/j.issn1002-2767.2023.08.0008

Xu SL, Li ZG, and Gong M. 2011. Protective action of exogenous betaine on seed germination and seedling growth of *Jatropha curcas* under peg stress. *Seed* 30(9):29-33. DOI: 10.3969/j.issn.1001-4705.2011.09.008

Yang SS, Weng MY, Dai M, Sun BF, and Shan L. 2008. Response of the osmotic stress on the wheat seedlings drought resistance under the different time betaine pretreatment. *Research of Soil and Water Conservation* 15(06):241-244. https://stbcyj.publish.founderss.cn/thesisDetails?columnId=76534309&Fpath=home&index=0&lang=zh

Yang SY, Zhang JX, Lu JL, and Shi RL. 2000. Studies on effect of exogenous betaine on physiological index of drought resistance for winter wheat. *Acta Botanica Boreali-Occidentalia Sinica* 20(6):1041-1045. DOI: 10.3321/j.issn:1000-4025.2000.06.024

Yang XY, Song T, Liu H, and Wang H. 2017. Effects of exogenous glycine betaine on the growth and chlorophyll content of maize seedlings under NaCl stress. *Hubei Agricultural Sciences* 56(5):830-833, 875. DOI: 10.14088/j.cnki.issn0439-8114.2017.05.008

Yang ZJ, Li GJ, Li X, Huang X, Liu FH, and Du GH. 2020. Physiological effects of different exogenous substances spraying on seedlings of ‘Yunma 1’ 'under drought stress. *Journal of Yunnan University(Natural Sciences Edition)* 42(2):374-381. DOI: 10.7540/j.ynu.20190301

Zhang EF, Li YY, and Hao WL. 2022. Effects of exogenous glycine betaine on seed germination and physiological characteristics of *Coriandrum sativum* seedlings under drought stress. *Seed* 41(12):93-97, 105. DOI: 10.16590/j.cnki.1001-4705.2022.12.093

Zhang HM, Li JJ, Hei GG, Cao RX, Zhou XY, Li LL, Yang WX, and Wu NB. 2014. Effects of exogenous betaine on the secondary metabolites of *Pinellia ternata* under drought stress. *Acta Prataculturae Sinica* 23(4):229-236. DOI: 10.11686/cyxb20140428

Zhang JX, Xu FL, Lu JL, Li YL, and Li D. 2003. Effect of exogenous betaine on drought resistance of different crops. *Agricultural Research in the Arid Areas* 21(2):86-90. DOI: 10.3321/j.issn:1000-7601.2003.02.020

Zhang L, Jia ZG, Liu XX, Fan CL, and Yuan HF. 2016. The effect of exogenous substances on the germination and physiological characteristics of ephedra seeds under drought stress. *Journal of Chinese Medicinal Materials* 39(11):2450-2455. DOI: 10.13863/j.issn1001-4454.2016.11.005

Zhang SG, Gao JY, Song JZ, and Weng YJ. 1998. The alleviating effect of betaine on salt damage during the growth process of wheat seedlings. *Beijing Agricultural Sciences* 16(03):14-18. https://kns.cnki.net/kcms2/article/abstract?v=QLLcG2LOr2qTgfn3fsNw0it_qm7l7UvrBvGvFiIFwlGPFuhLdZNMMIg-GbH3iFGjxxfgG1h6B41IBg54_eGkjEaD-d6Q3ppE9BRa6Urfkdb9qPG4yNPuJRrCpd5DUFV1MxEzhx-V4LVe5ocrBWwJ_rauVU6PdgwYLwexP9l5h4g=&uniplatform=NZKPT&language=CHS

Zhang SG, Gao JY, and Song JZ. 1999a. Effects of betaine on content and distribution of Na^+^, K^+^ and Cl^-^ in wheat( *Triticum aestivum* L.) Seedling under salt stress. *Acta Bot. Boreal. Occident. Sin.* 19(02):112-117. http://xbzwxb.cnjournals.com/xbzwxb/article/abstract/19990233?st=article_issue

Zhang SG, Gao JY, and Song JZ. 1999b. Effects of betaine on activities of membrane protective enzymes in wheat(*Triticum aestivum* L. ) Seedlings under NaCl stress. *Chinese Bulletin of Botany* 16(4):429. DOI: 10.3969/j.issn.1674-3466.1999.04.018

Zhang SG, Gao JY, and Song JZ. 2000. Effects of exogenous betaine on the contents of some resistance-related substances the absorption and transportation of Na^+^, K^+^ in wheat seedlings under NaCl stress. *Plant Physiology Communications* 36(1):23-26. DOI: 10.13592/j.cnki.ppj.2000.01.007

Zhang X, Zhang FX, Sun QT, Zhang Y, and Han TJ. 2014. Preliminary report on the prevention of cherry cracking by exogenous betaine. *Yantai Fruits* 1(1):10-11. DOI: 10.3969/j.issn.1005-9938.2014.01.005

Zhang YF, Mo LL, Niu YF, Peng SJ, Wang DK, and Geng KY. 2016. Physiological mechanism of exogenous betaine and calcium chloride on improving cold tolerance in *Duranta repens*. *Northern Horticulture* 40(8):154-159. DOI: 10.11937/bfyy.201608042

Zhao BS, Yi YJ, Liu JY, and Liu JY. 2001. Exogenous betaine improves the growth and photo-synthesis of wheat seedlings under drought/salt stress. *Chinese Bulletin of Botany* 18(3):378-380, 366. DOI: 10.3969/j.issn.1674-3466.2001.03.019

Zhao H, Cao YY, Zhou R, and Ji BH. 2006. Exogenous glycinebetaine improves photosynthesis of rice seedlings under salt stress. *Acta Agriculturae Boreali-Sinica* 21(6):72-74. DOI: 10.3321/j.issn:1000-7091.2006.06.018

Zhao HY, Hai MR, Da BXLT, Lu P, Guo DY, and Zhou XW. 2010. Effects of exogenous osmotic substances on growth and physiological characteristics of *Cucumis sativus* L. under NaCl stress. *Journal of Anhui Agri. Sci.* 38(19):10004-10006. DOI: 10.13989/j.cnki.0517-6611.2010.19.115

Zhao JL, and Niu GY. 2009. Effects of exogenous glycinebetaine on salt resistance of cucumber. *Journal of Henan Agricultural Sciences* 38(6):112-115. DOI: 10.3969/j.issn.1004-3268.2009.06.029

Zhao XQ, Fang P, Peng YL, and Zhang JW. 2020. Physiological response of different maize inbred lines to exogenous GB under low-temperature stress. *Agricultural Research in the Arid Areas* 38(3):81-88. DOI: 10.7606/j.issn.1000-7601.2020.03.11

Zhao XQ, Peng YL, Fang P, Wu BY, and Yan HP. 2018. Mitigation effect analysis of different exogenous regulatory substances on maize under low-temperature stress. *Agricultural Research in the Arid Areas* 36(3):184-193, 229. DOI: 10.7606/j.issn.1000-7601.2018.03.28

Zheng GP, Wang XY, Liu XL, Tian ZR, Wang C, and Ji WQ. 2019. The role of lipoic acid and betaine in wheat tissue culture. *Journal of Triticeae Crops* 39(3):308-313. DOI: 10.7606/j.issn.1009-1041.2019.03.08

Zheng YF, Zhang XM, and Zhao JH. 2018. The effects of four different exogenous substances on the germination of *Primula vularis* seeds under drought stress. *Jiangsu Agricultural Sciences* 46(7):155-158. DOI: 10.15889/j.issn.1002-1302.2018.07.038

Zhong GH, and Wang JL. 1997a. The protective effect of the external betaine on the leaves of *Brassica pekinensis* under NaCl stress. *Plant Physiology Communications* 33(5):333-335. DOI: 10.13592/j.cnki.ppj.1997.05.003

Zhong GH, and Wang JL. 1997b. The inhibitory effect of exogenous betaine on leaf senescence of *Hordeum vulgare* L. *Barley and Cereal Sciences* 14(02):21-23. DOI: 10.14069/j.cnki.32-1769/s.1997.02.008

Zhong XJ, Li Q, Zhou XX, Liu JL, Li MC, and Cheng CX. 2017. Effects of three exogenous plant growth regulators on tobacco physiology under drought stress. *Journal of Anhui Agricultural University* 44(06):1139-1143. DOI: 10.13610/j.cnki.1672-352x.20171214.035

Zhou QC, Hua C, Zhou F, Chen AJ, and Wang RL. 2009. Effects of exogenous GB on germination and seedlings of *Salicornia europaea* L. and *Salicornia bigelovii* Torr. under NaCl stress. *Agricultural Research in the Arid Areas* 27(6):179-184. DOI: 10.7606/j.issn.1000-7601.2009.06.35

Zhou R, and Zhao H. 2008. Effects of betaine on photosynthesis of tomato under salt stress. *Journal of Anhui Agricultural Sciences* 36(33):14406-14407. DOI: 10.3969/j.issn.0517-6611.2008.33.022
